# Supplementary material for: The termination of UHRF1-dependent PAF15 ubiquitin signaling is regulated by USP7 and ATAD5
Source: eLife. 2023 Feb 3;12:e79013. doi: 10.7554/eLife.79013 (PMC9943068; doi:10.7554/eLife.79013)
Supplement: Figure 1—source data 1. [file elife-79013-fig1-data1.zip › Figure 1-source data/Figure 1-Source Data.pptx]

## Slide 1
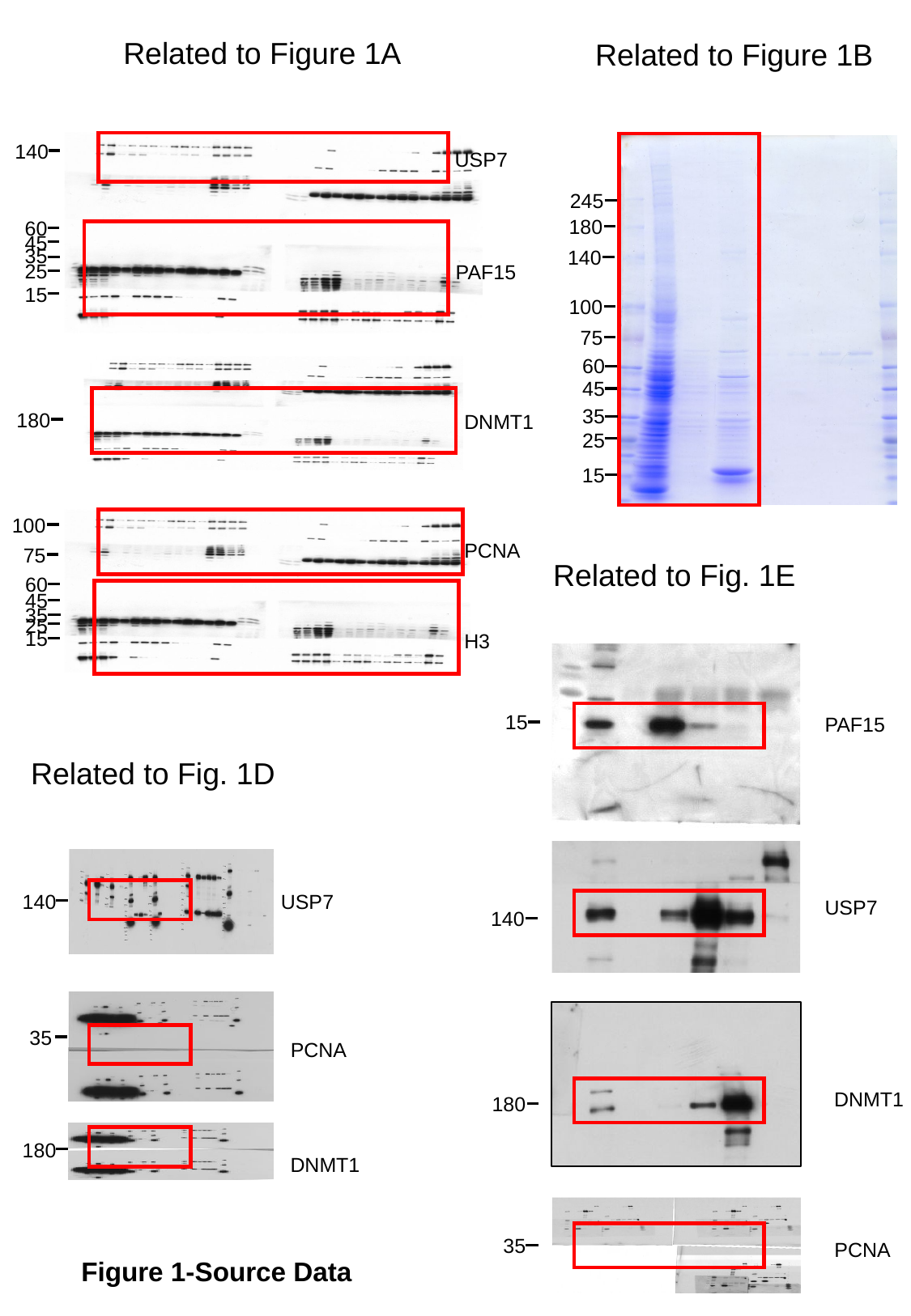

Related to Figure 1A
Related to Figure 1B
140
USP7
245
180
60
45
35
140
25
PAF15
15
100
75
60
45
35
180
DNMT1
25
15
100
PCNA
75
Related to Fig. 1E
60
45
35
25
15
H3
15
PAF15
Related to Fig. 1D
140
USP7
USP7
140
35
PCNA
DNMT1
180
180
DNMT1
35
PCNA
Figure 1-Source Data
