# Supplementary material for: The termination of UHRF1-dependent PAF15 ubiquitin signaling is regulated by USP7 and ATAD5
Source: eLife. 2023 Feb 3;12:e79013. doi: 10.7554/eLife.79013 (PMC9943068; doi:10.7554/eLife.79013)
Supplement: Figure 1—figure supplement 1—source data 1. [file elife-79013-fig1-figsupp1-data1.zip › Figure 1-figure supplement 1-source data/Figure1 - figure supplement 1-Source Data.pptx]

## Slide 1
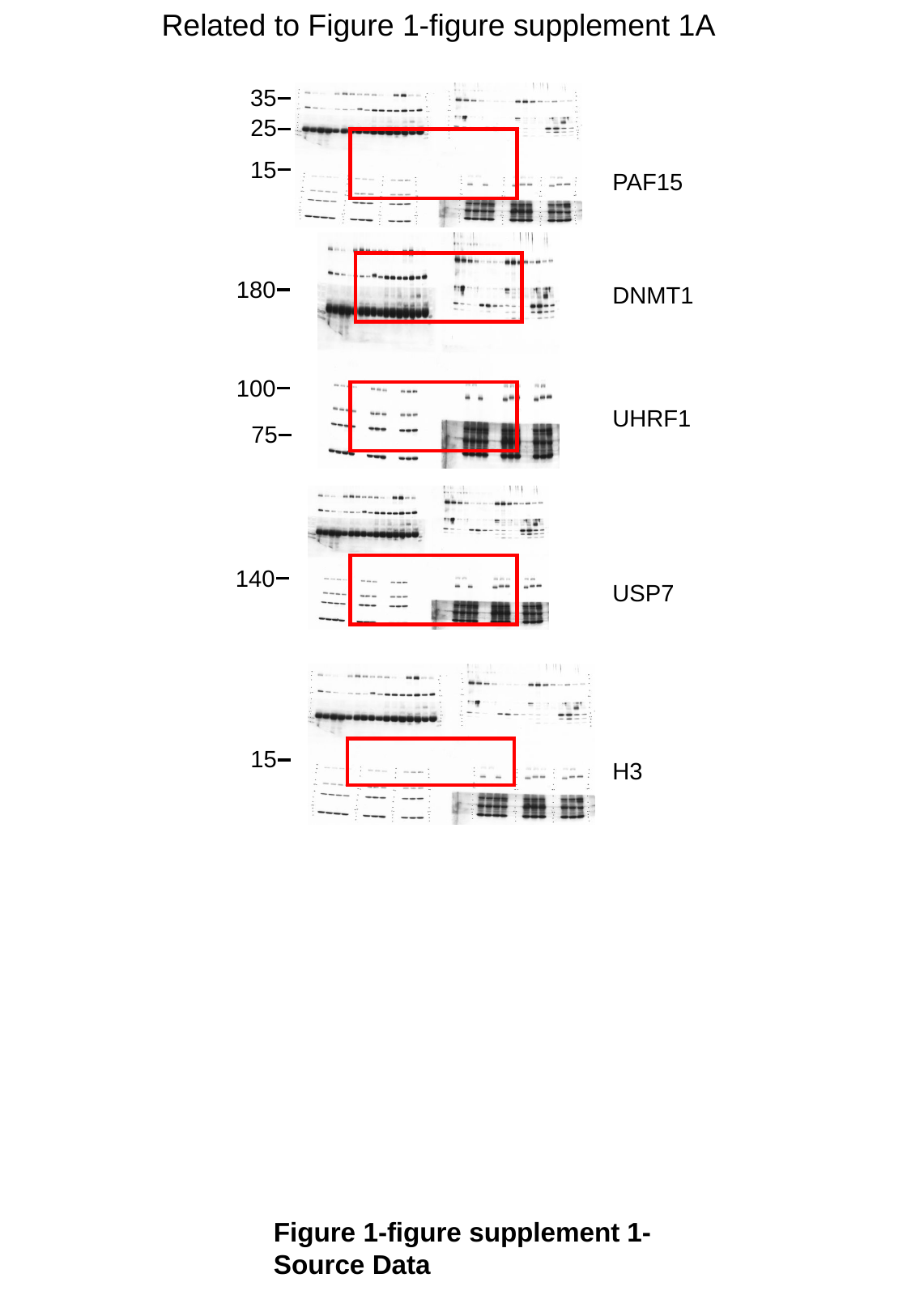

Related to Figure 1-figure supplement 1A
35
25
15
PAF15
180
DNMT1
100
UHRF1
75
140
USP7
15
H3
Figure 1-figure supplement 1-Source Data

## Slide 2
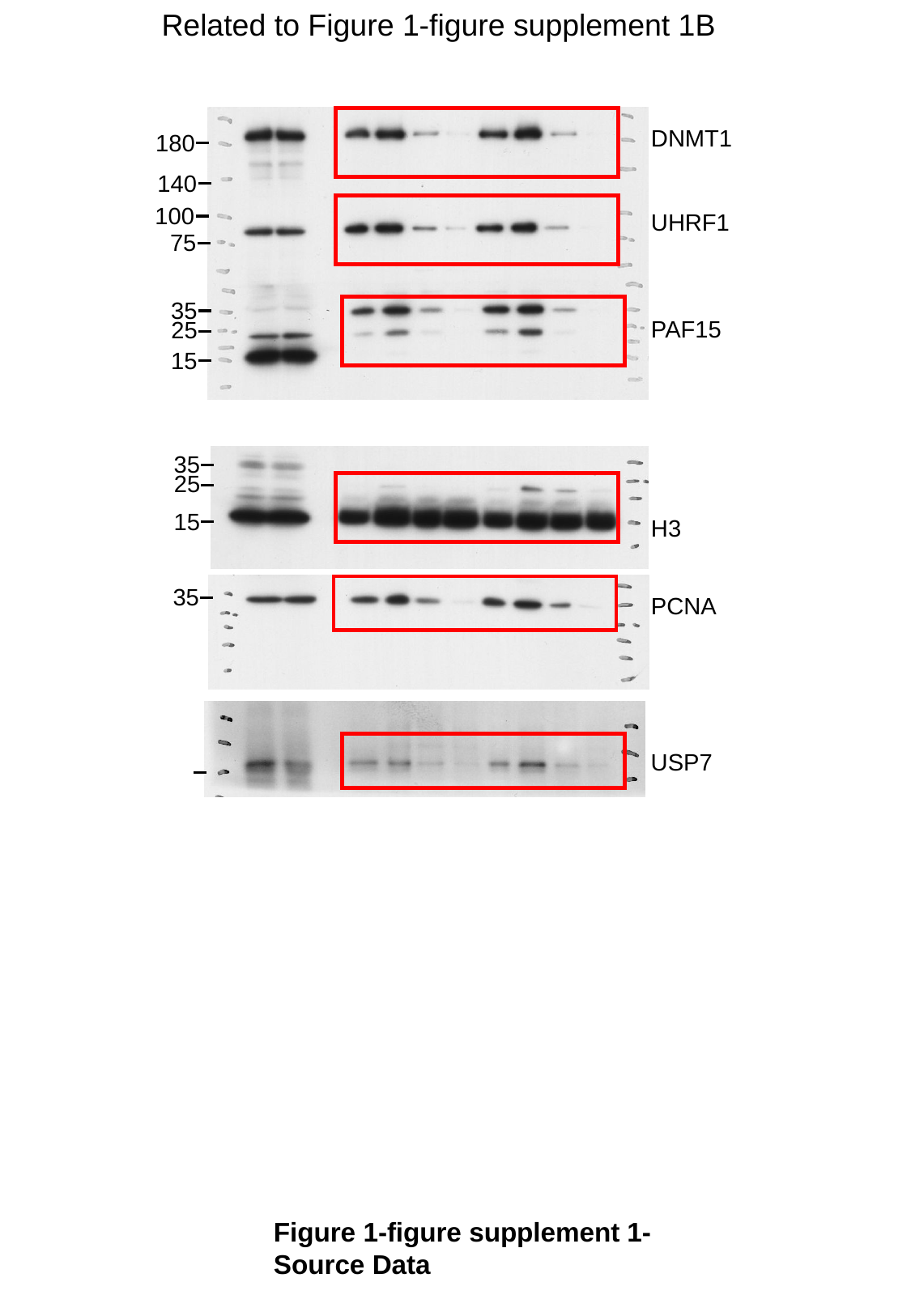

Related to Figure 1-figure supplement 1B
DNMT1
180
140
100
UHRF1
75
35
PAF15
25
15
35
25
15
H3
35
PCNA
USP7
Figure 1-figure supplement 1-Source Data

## Slide 3
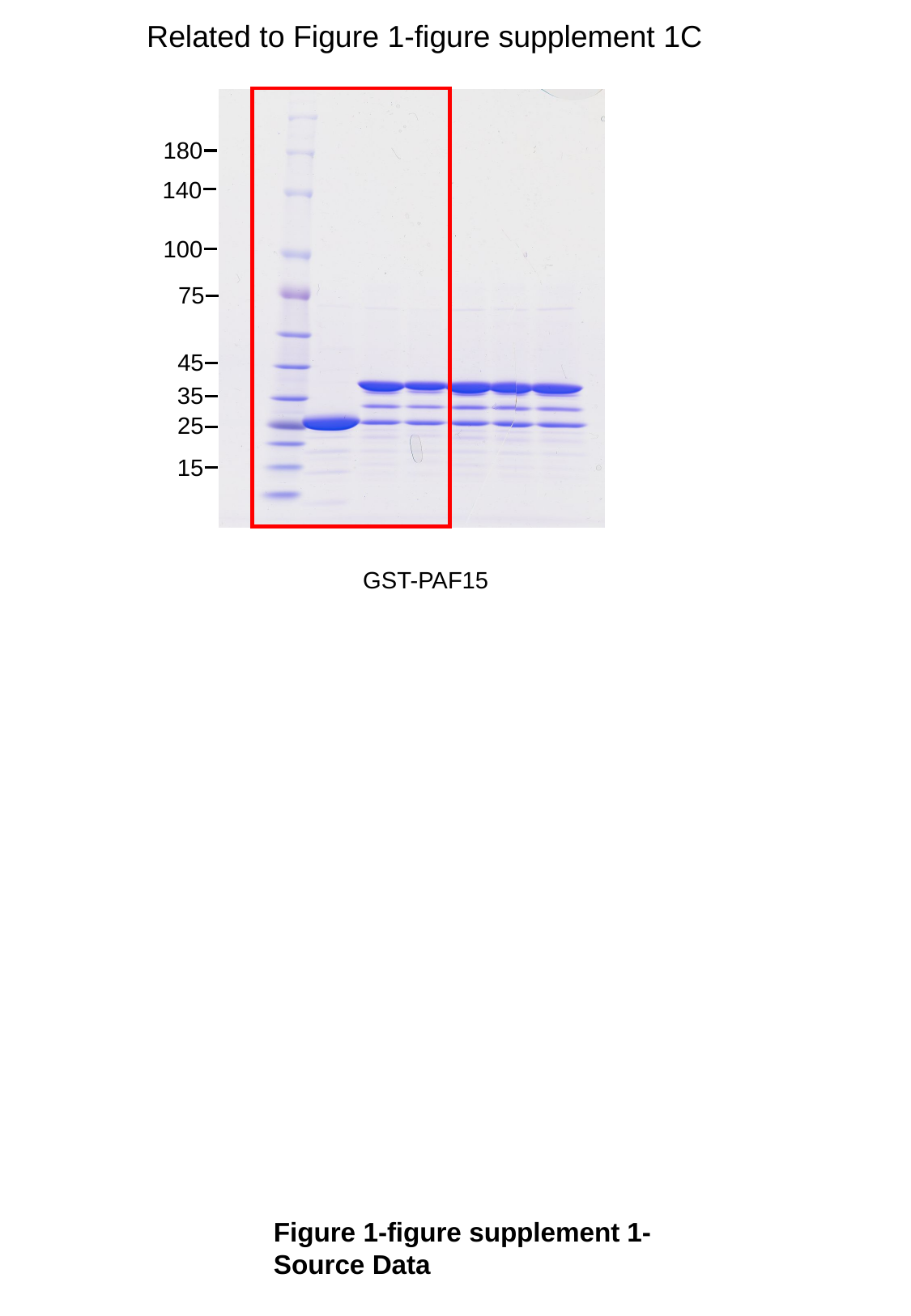

Related to Figure 1-figure supplement 1C
180
140
100
75
45
35
25
15
GST-PAF15
Figure 1-figure supplement 1-Source Data

## Slide 4
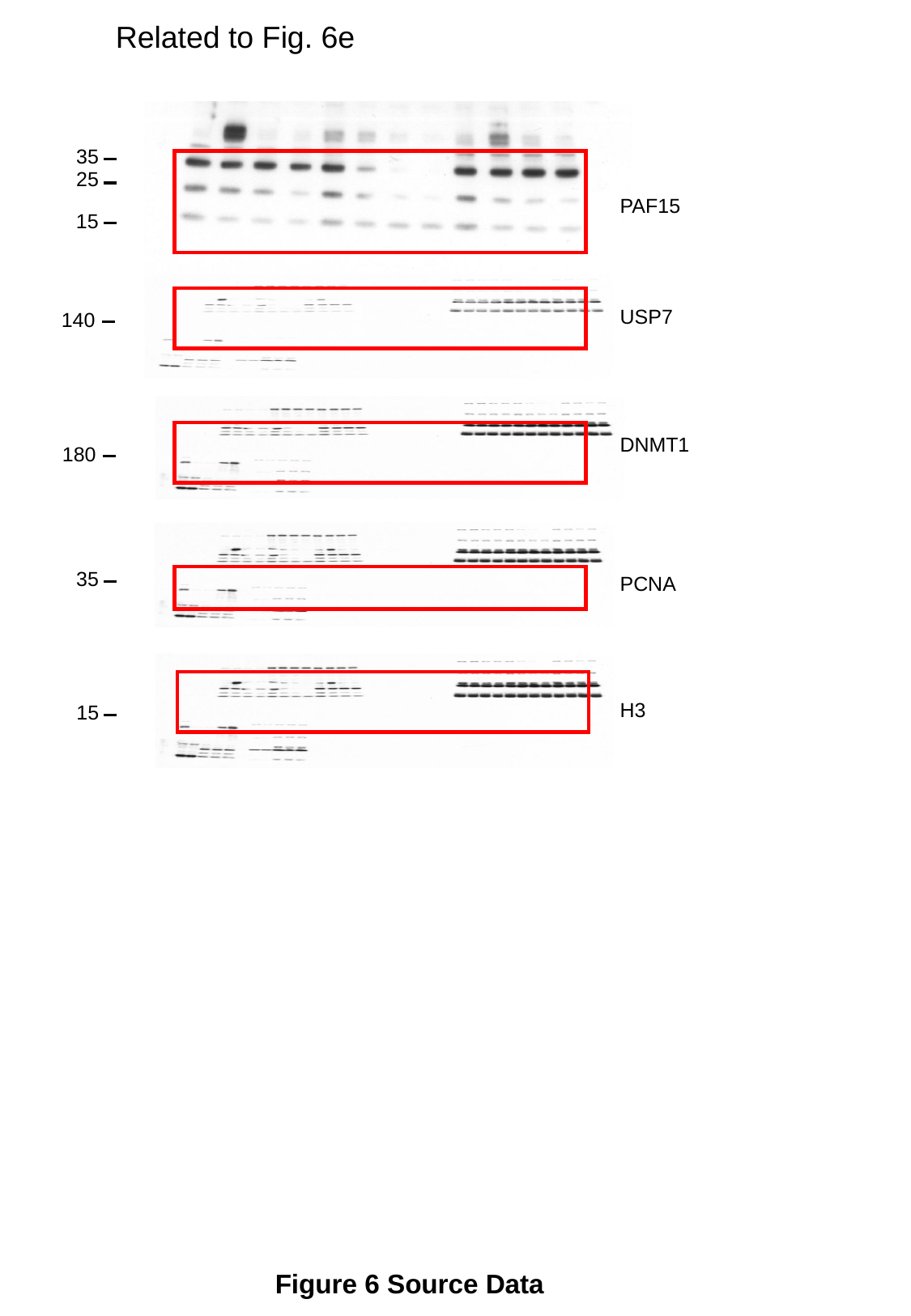

Related to Fig. 6e
35
25
PAF15
15
USP7
140
DNMT1
180
35
PCNA
H3
15
Figure 6 Source Data

## Slide 5
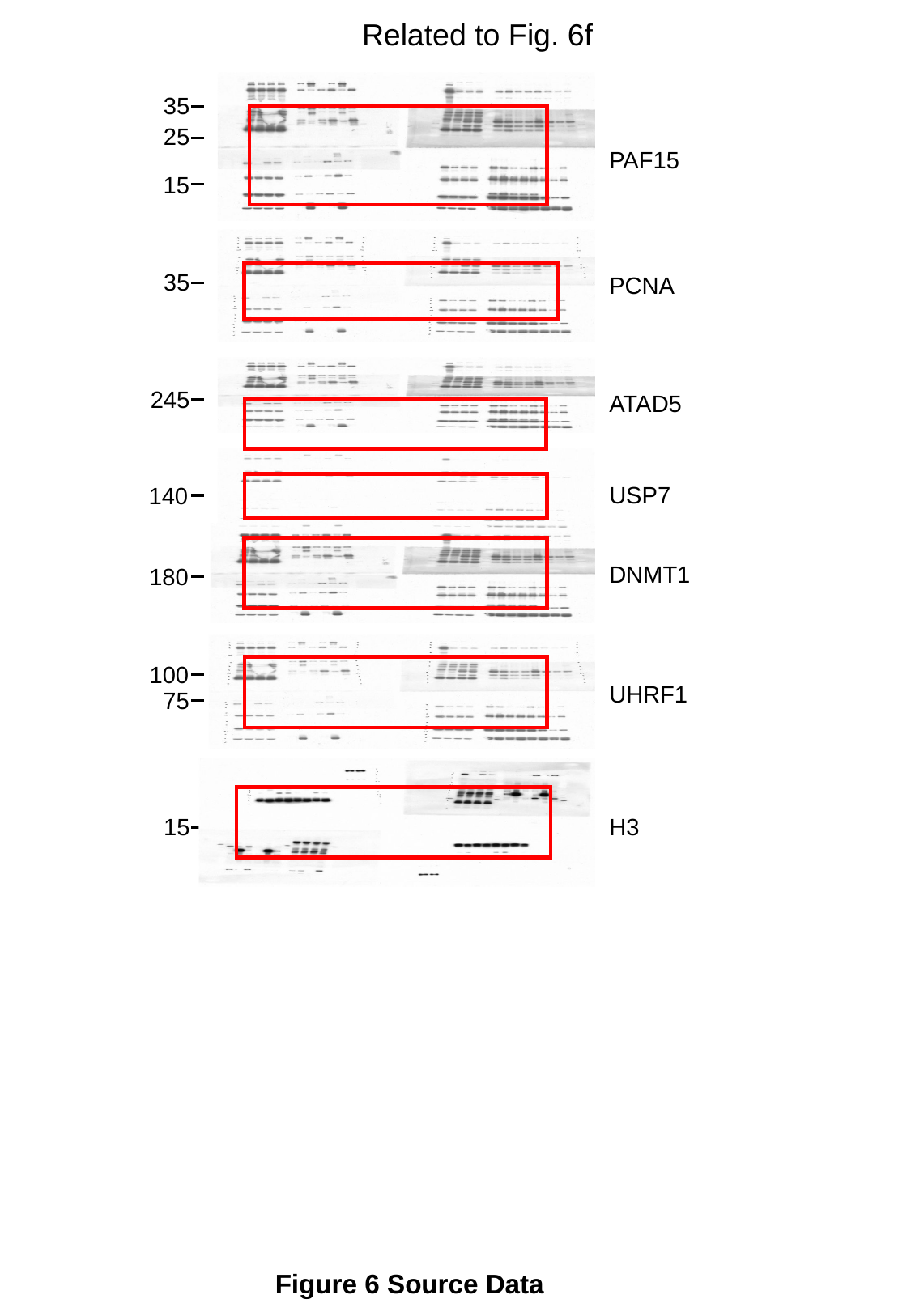

Related to Fig. 6f
35
25
PAF15
15
35
PCNA
245
ATAD5
USP7
140
DNMT1
180
100
UHRF1
75
H3
15
Figure 6 Source Data
