# Supplementary material for: The termination of UHRF1-dependent PAF15 ubiquitin signaling is regulated by USP7 and ATAD5
Source: eLife. 2023 Feb 3;12:e79013. doi: 10.7554/eLife.79013 (PMC9943068; doi:10.7554/eLife.79013)
Supplement: Figure 2—source data 1. [file elife-79013-fig2-data1.zip › Figure 2-source data/Figure 2-Source Data.pptx]

## Slide 1
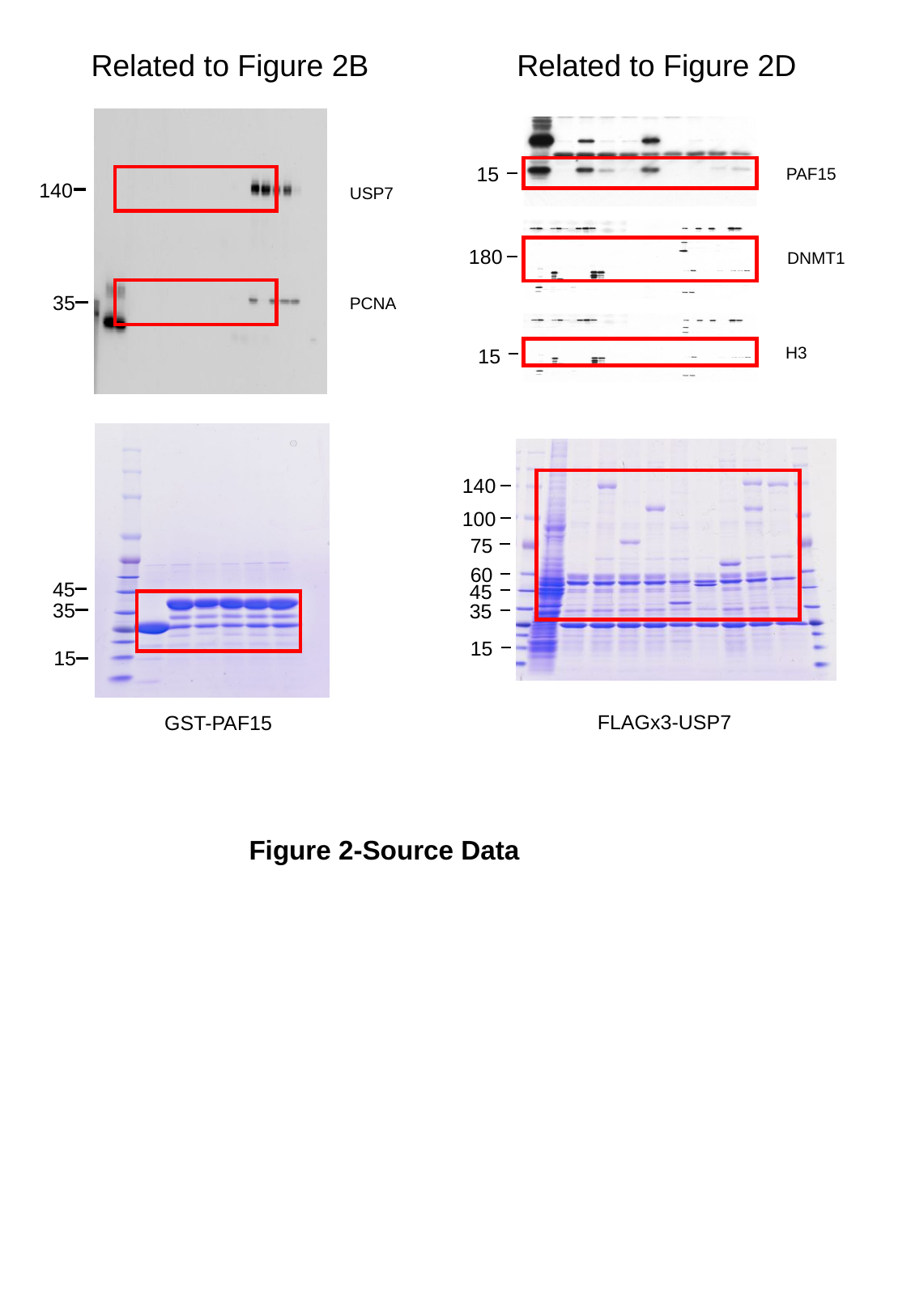

Related to Figure 2B
Related to Figure 2D
15
PAF15
140
USP7
180
DNMT1
35
PCNA
H3
15
140
100
75
60
45
45
35
35
15
15
FLAGx3-USP7
GST-PAF15
Figure 2-Source Data
