# Supplementary material for: The termination of UHRF1-dependent PAF15 ubiquitin signaling is regulated by USP7 and ATAD5
Source: eLife. 2023 Feb 3;12:e79013. doi: 10.7554/eLife.79013 (PMC9943068; doi:10.7554/eLife.79013)
Supplement: Figure 3—source data 1. [file elife-79013-fig3-data1.zip › Figure 3-source data/Figure 3-Source Data.pptx]

## Slide 1
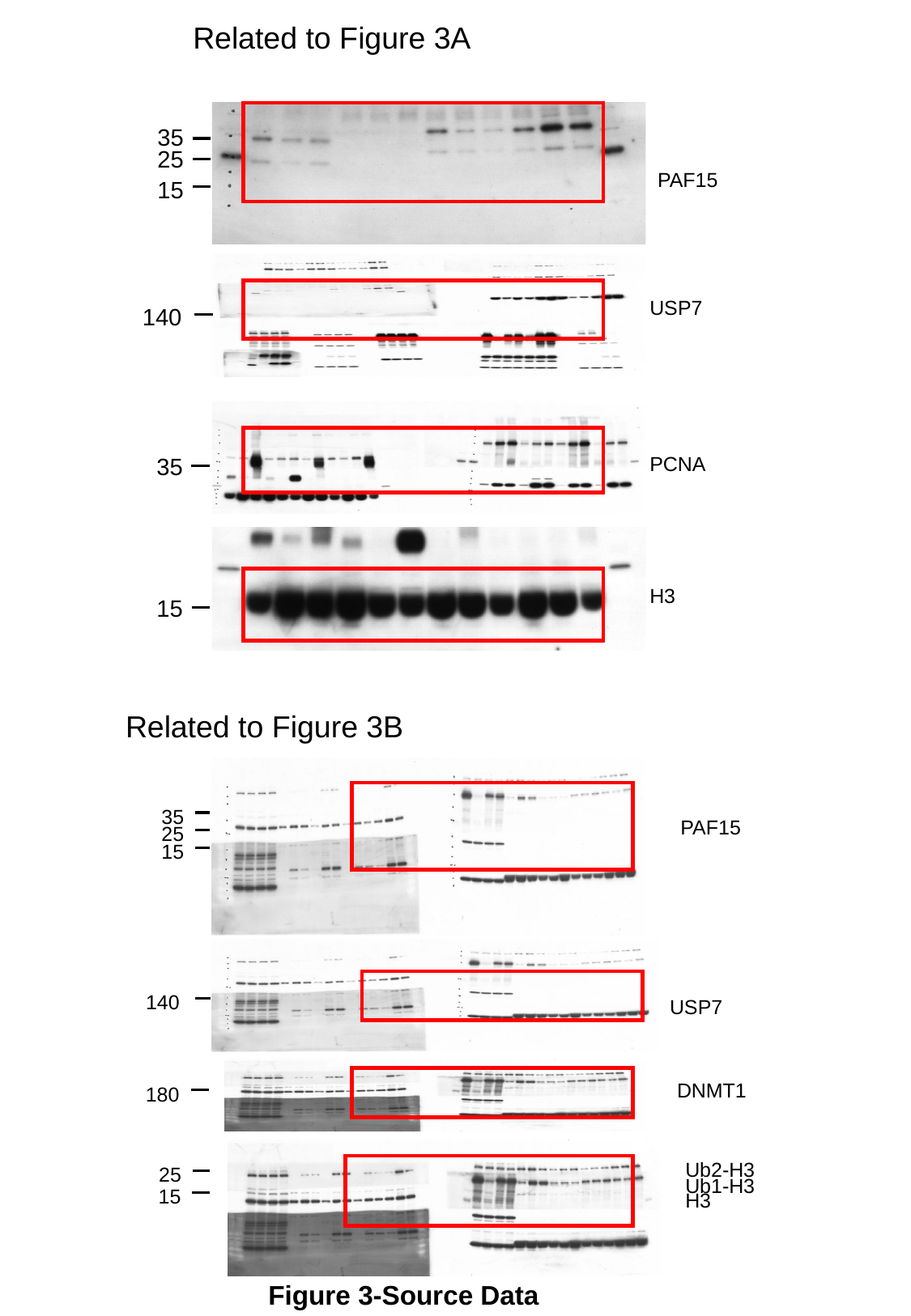

Related to Figure 3A
35
25
PAF15
15
USP7
140
PCNA
35
H3
15
Related to Figure 3B
35
PAF15
25
15
140
USP7
DNMT1
180
Ub2-H3
25
Ub1-H3
15
H3
Figure 3-Source Data

## Slide 2
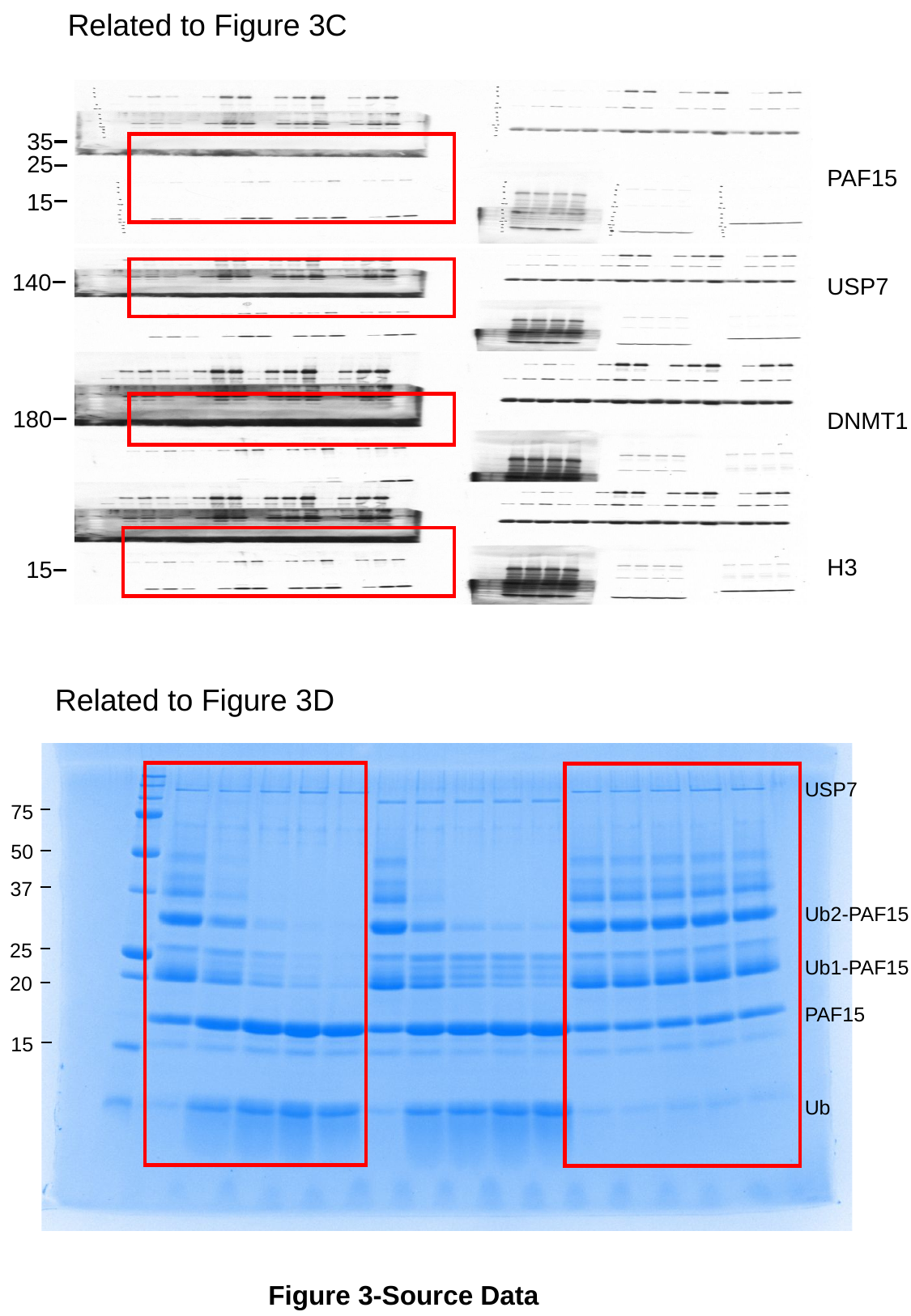

Related to Figure 3C
35
25
PAF15
15
140
USP7
180
DNMT1
H3
15
Related to Figure 3D
USP7
75
50
37
Ub2-PAF15
25
Ub1-PAF15
20
PAF15
15
Ub
Figure 3-Source Data
