# Supplementary material for: The termination of UHRF1-dependent PAF15 ubiquitin signaling is regulated by USP7 and ATAD5
Source: eLife. 2023 Feb 3;12:e79013. doi: 10.7554/eLife.79013 (PMC9943068; doi:10.7554/eLife.79013)
Supplement: Figure 3—figure supplement 1—source data 1. [file elife-79013-fig3-figsupp1-data1.zip › Figure 3-figure supplment 1-source data/Figure3- figure supplement 1-Source Data.pptx]

## Slide 1
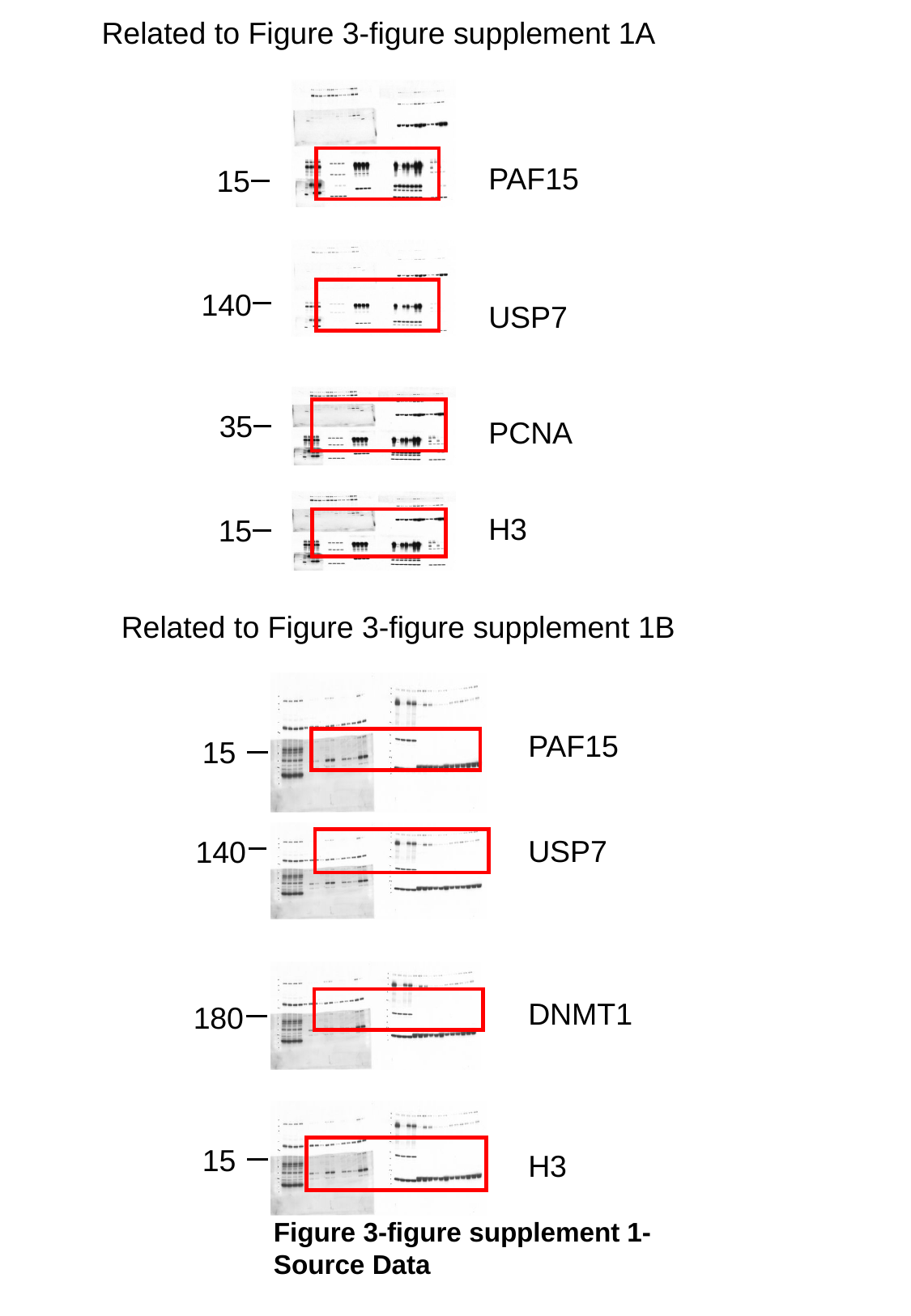

Related to Figure 3-figure supplement 1A
PAF15
15
140
USP7
35
PCNA
H3
15
Related to Figure 3-figure supplement 1B
PAF15
15
USP7
140
DNMT1
180
15
H3
Figure 3-figure supplement 1-Source Data

## Slide 2
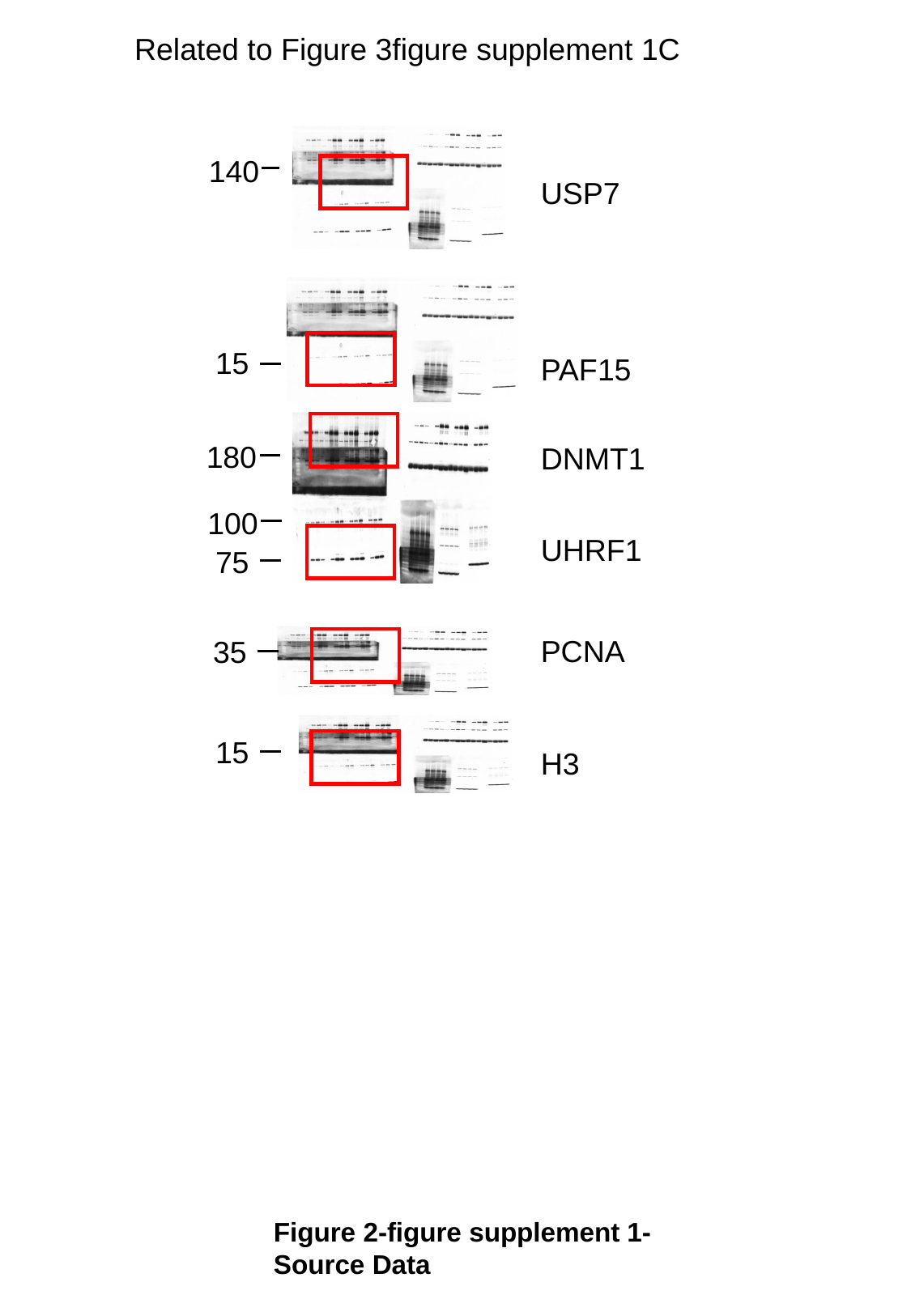

Related to Figure 3figure supplement 1C
140
USP7
15
PAF15
180
DNMT1
100
UHRF1
75
PCNA
35
15
H3
Figure 2-figure supplement 1-Source Data

## Slide 3
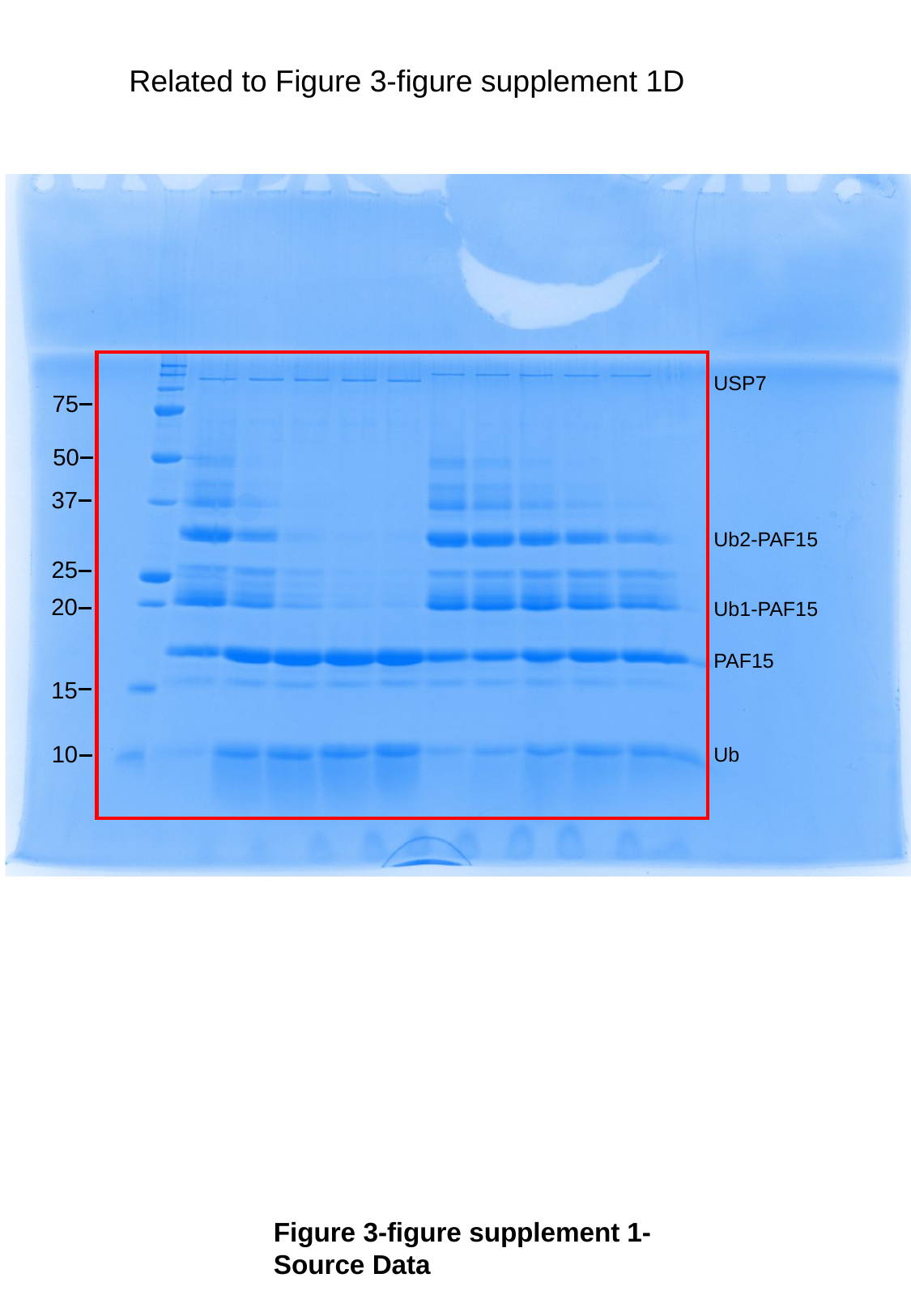

Related to Figure 3-figure supplement 1D
USP7
75
50
37
Ub2-PAF15
25
20
Ub1-PAF15
PAF15
15
10
Ub
Figure 3-figure supplement 1-Source Data
