# Supplementary material for: The termination of UHRF1-dependent PAF15 ubiquitin signaling is regulated by USP7 and ATAD5
Source: eLife. 2023 Feb 3;12:e79013. doi: 10.7554/eLife.79013 (PMC9943068; doi:10.7554/eLife.79013)
Supplement: Figure 4—source data 1. [file elife-79013-fig4-data1.zip › Figure 4-source data/Figure 4-Source Data.pptx]

## Slide 1
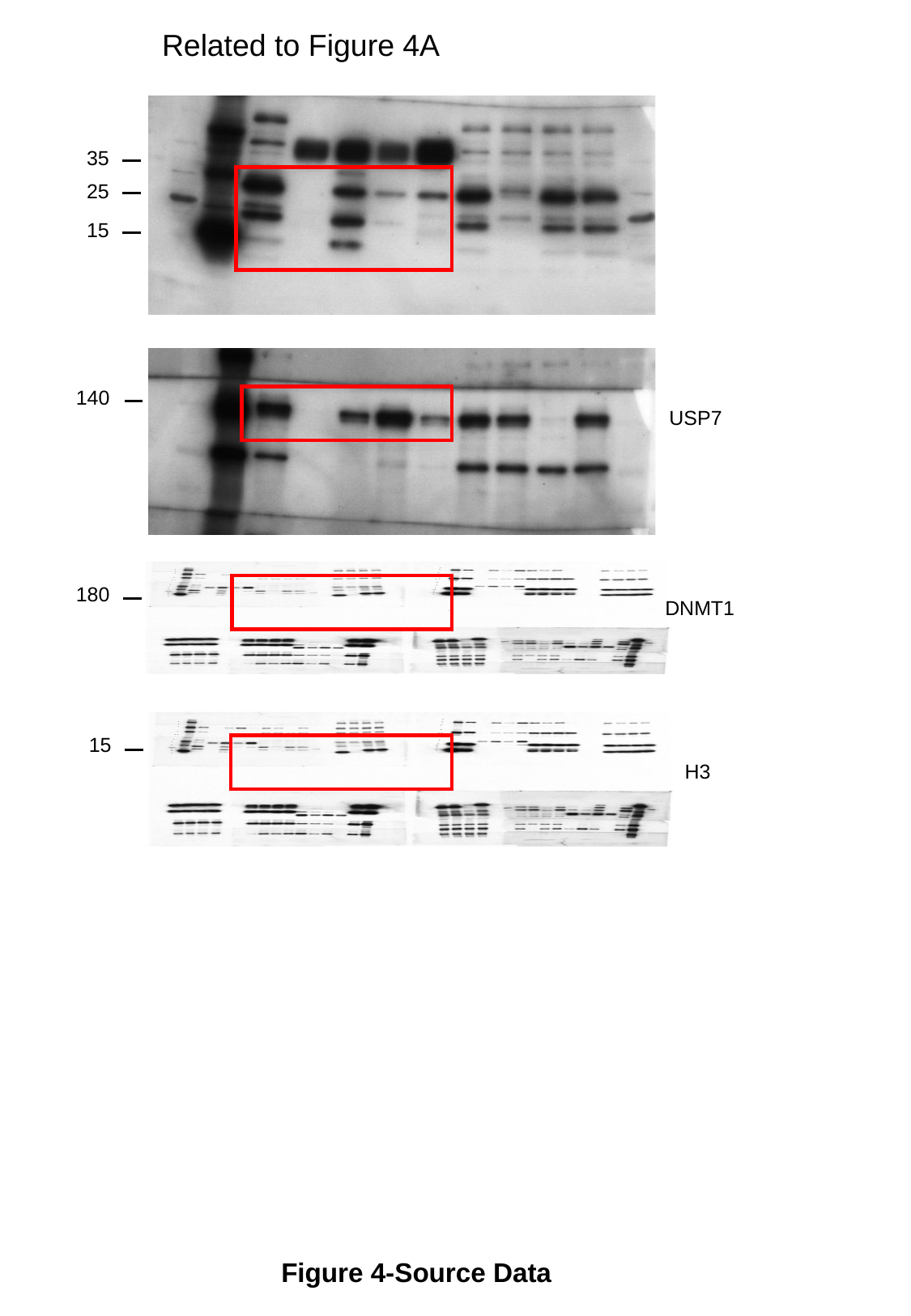

Related to Figure 4A
35
25
15
140
USP7
180
DNMT1
15
H3
Figure 4-Source Data

## Slide 2
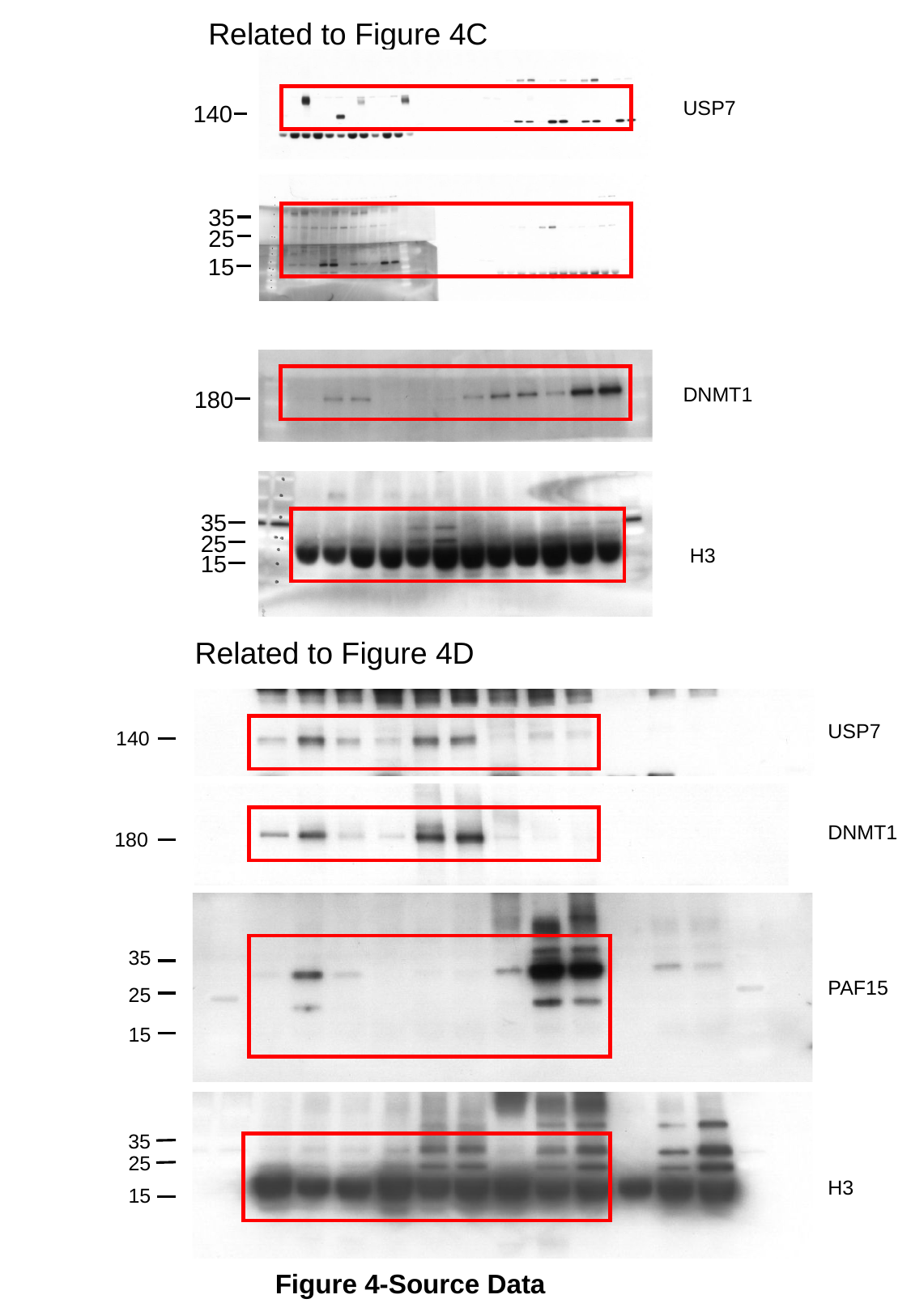

Related to Figure 4C
USP7
140
35
25
15
DNMT1
180
35
25
H3
15
Related to Figure 4D
USP7
140
DNMT1
180
35
PAF15
25
15
35
25
H3
15
Figure 4-Source Data
