# Supplementary material for: The termination of UHRF1-dependent PAF15 ubiquitin signaling is regulated by USP7 and ATAD5
Source: eLife. 2023 Feb 3;12:e79013. doi: 10.7554/eLife.79013 (PMC9943068; doi:10.7554/eLife.79013)
Supplement: Figure 4—figure supplement 1—source data 1. [file elife-79013-fig4-figsupp1-data1.zip › Figure 4-figure supplement 1-source data/Figure4- figure supplement 1-Source Data.pptx]

## Slide 1
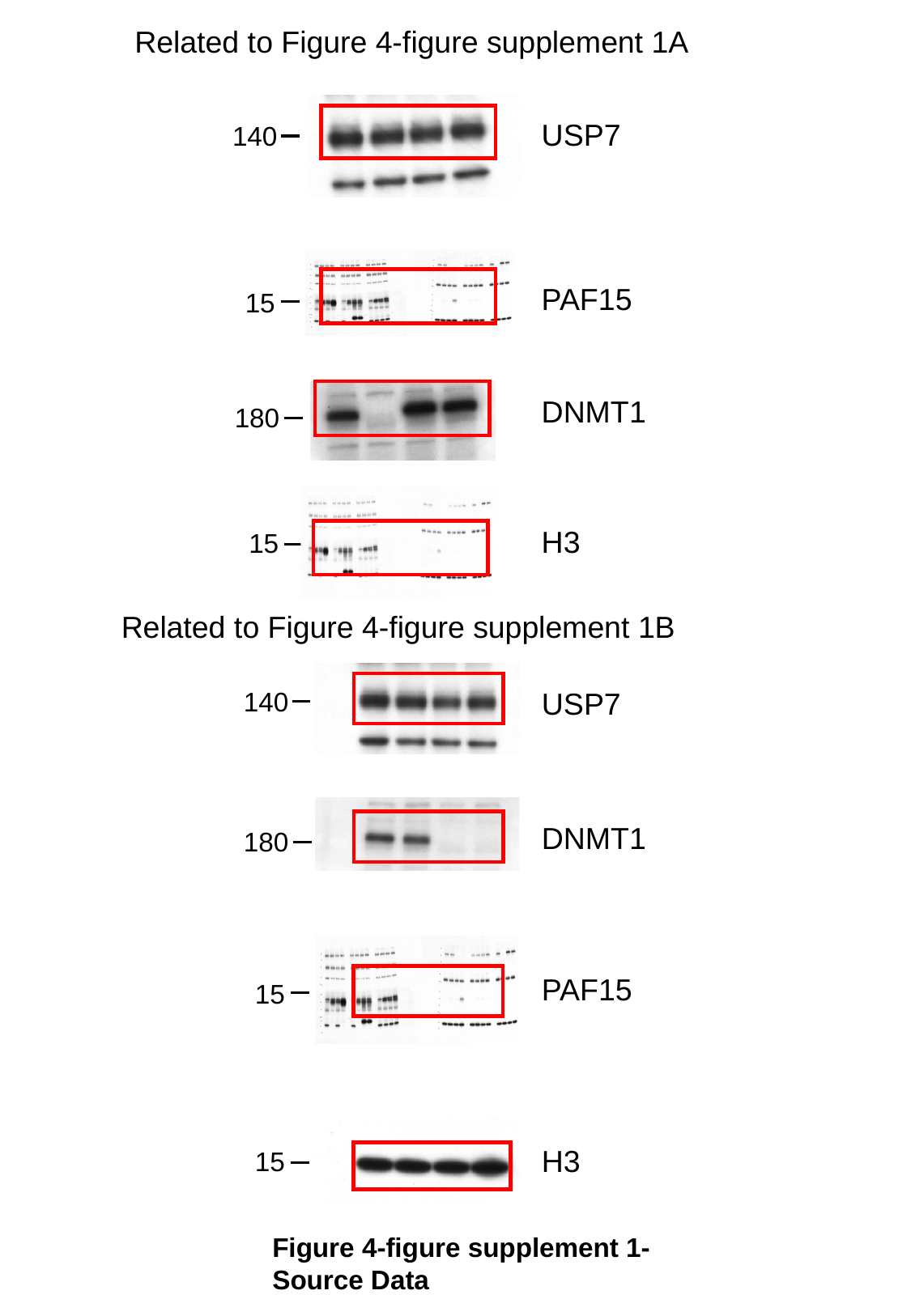

Related to Figure 4-figure supplement 1A
USP7
140
PAF15
15
DNMT1
180
H3
15
Related to Figure 4-figure supplement 1B
USP7
140
DNMT1
180
PAF15
15
H3
15
Figure 4-figure supplement 1-Source Data
