# Supplementary material for: The termination of UHRF1-dependent PAF15 ubiquitin signaling is regulated by USP7 and ATAD5
Source: eLife. 2023 Feb 3;12:e79013. doi: 10.7554/eLife.79013 (PMC9943068; doi:10.7554/eLife.79013)
Supplement: Figure 5—figure supplement 1—source data 1. [file elife-79013-fig5-figsupp1-data1.zip › Figure 5-figure supplement 1-source data/Figure5- figure supplement 1-Source Data.pptx]

## Slide 1
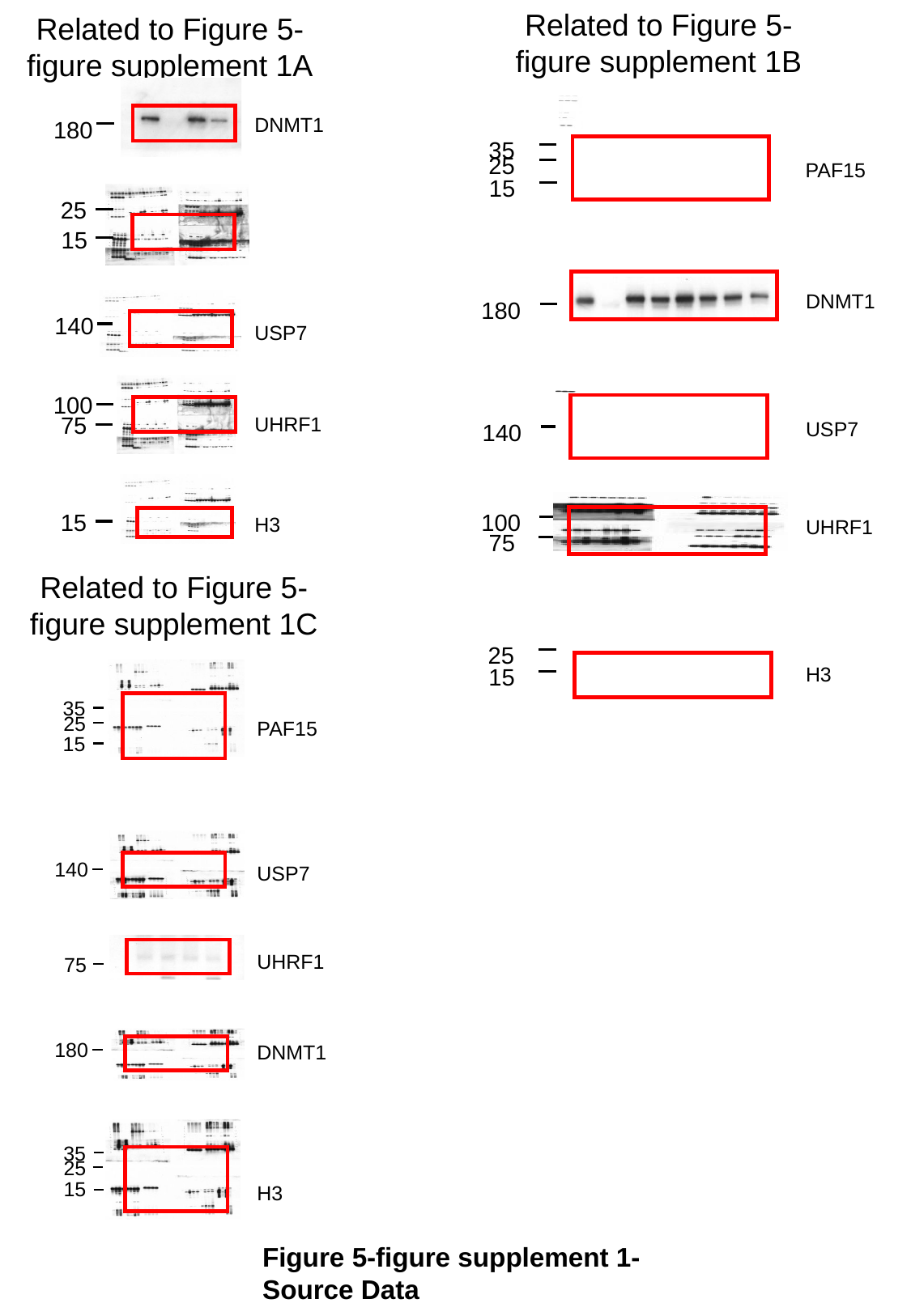

Related to Figure 5-
figure supplement 1B
Related to Figure 5-
figure supplement 1A
DNMT1
180
35
25
PAF15
15
25
15
DNMT1
180
140
USP7
100
75
UHRF1
USP7
140
100
15
H3
UHRF1
75
Related to Figure 5-
figure supplement 1C
25
H3
15
35
25
PAF15
15
140
USP7
UHRF1
75
180
DNMT1
35
25
15
H3
Figure 5-figure supplement 1-Source Data
