# Supplementary material for: The termination of UHRF1-dependent PAF15 ubiquitin signaling is regulated by USP7 and ATAD5
Source: eLife. 2023 Feb 3;12:e79013. doi: 10.7554/eLife.79013 (PMC9943068; doi:10.7554/eLife.79013)
Supplement: Figure 6—source data 1. [file elife-79013-fig6-data1.zip › Figure 6-source data/Figure 6-Source Data.pptx]

## Slide 1
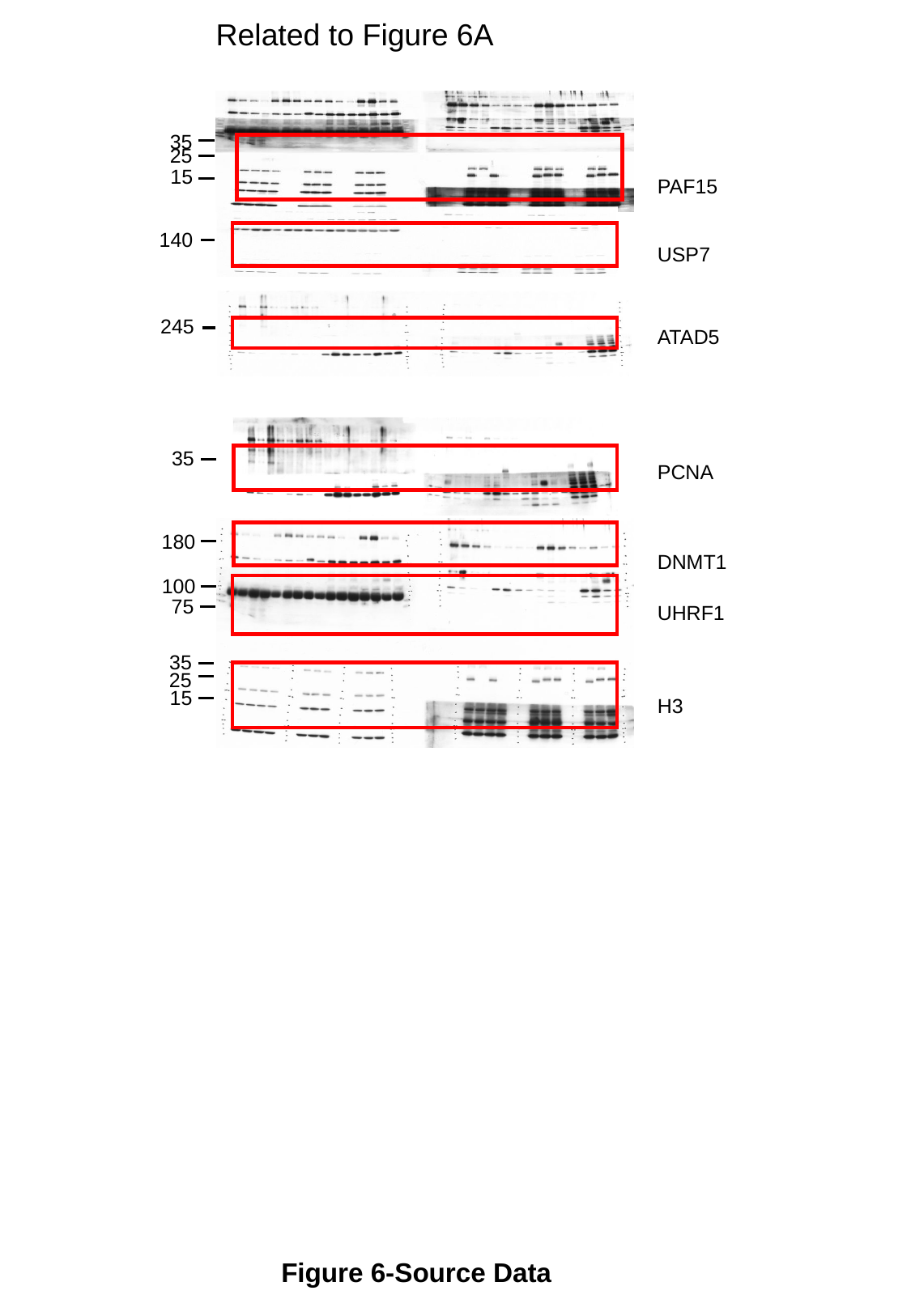

Related to Figure 6A
35
25
15
PAF15
140
USP7
245
ATAD5
35
PCNA
180
DNMT1
100
75
UHRF1
35
25
15
H3
Figure 6-Source Data

## Slide 2
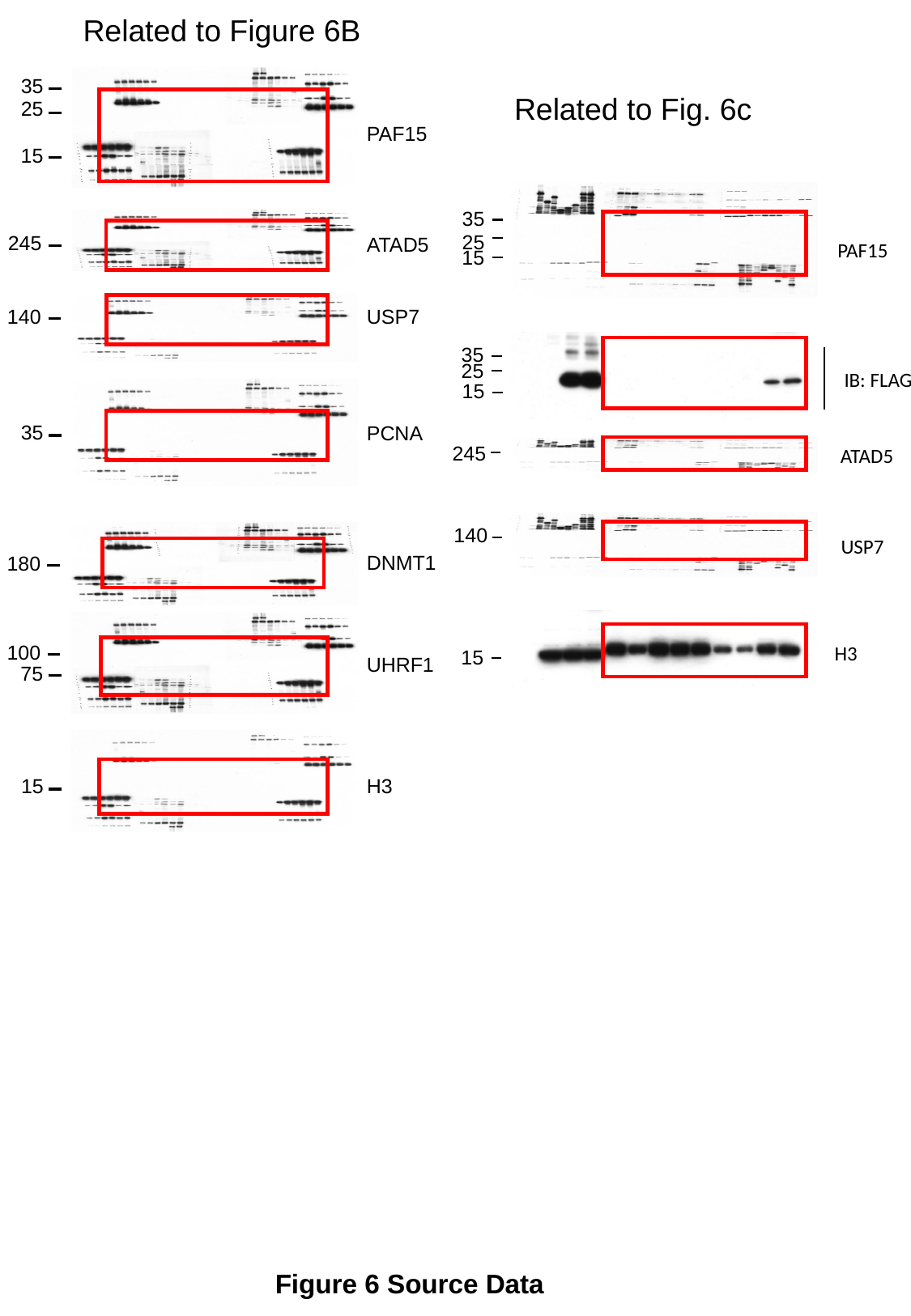

Related to Figure 6B
35
Related to Fig. 6c
25
PAF15
15
35
25
245
ATAD5
PAF15
15
USP7
140
35
25
IB: FLAG
15
35
PCNA
245
ATAD5
140
USP7
DNMT1
180
100
H3
15
UHRF1
75
H3
15
Figure 6 Source Data

## Slide 3
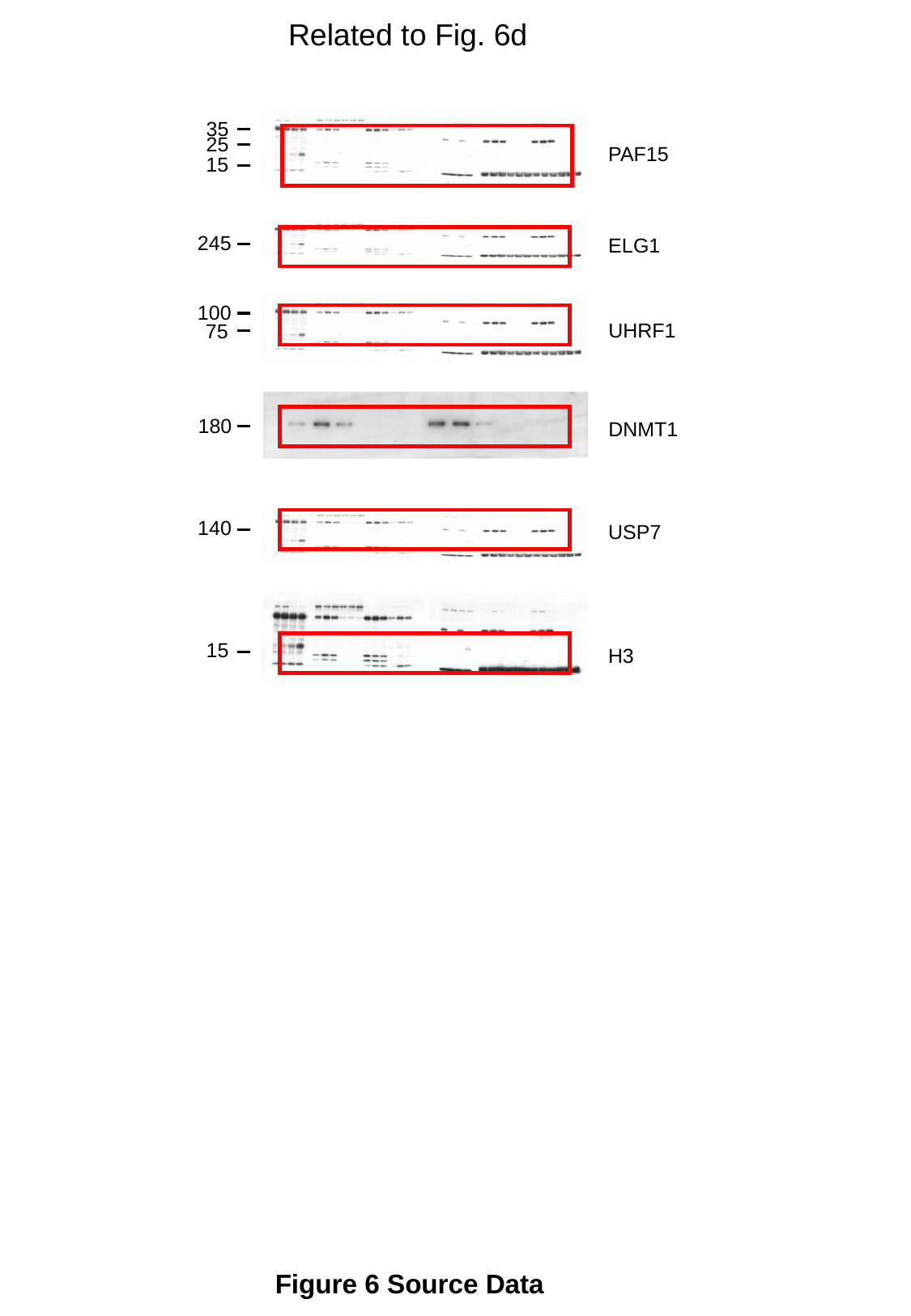

Related to Fig. 6d
35
25
PAF15
15
245
ELG1
100
UHRF1
75
180
DNMT1
140
USP7
15
H3
Figure 6 Source Data

## Slide 4
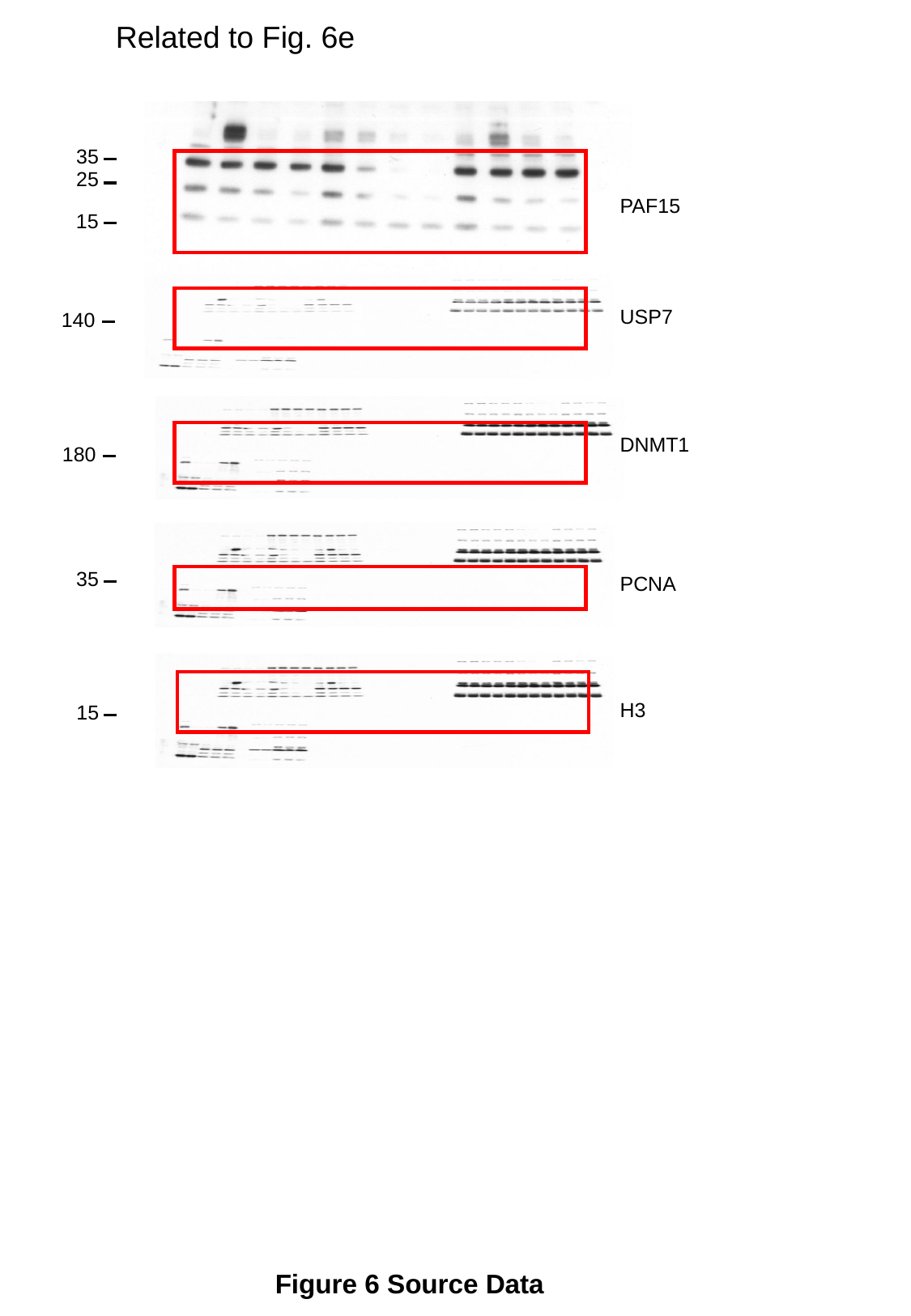

Related to Fig. 6e
35
25
PAF15
15
USP7
140
DNMT1
180
35
PCNA
H3
15
Figure 6 Source Data

## Slide 5
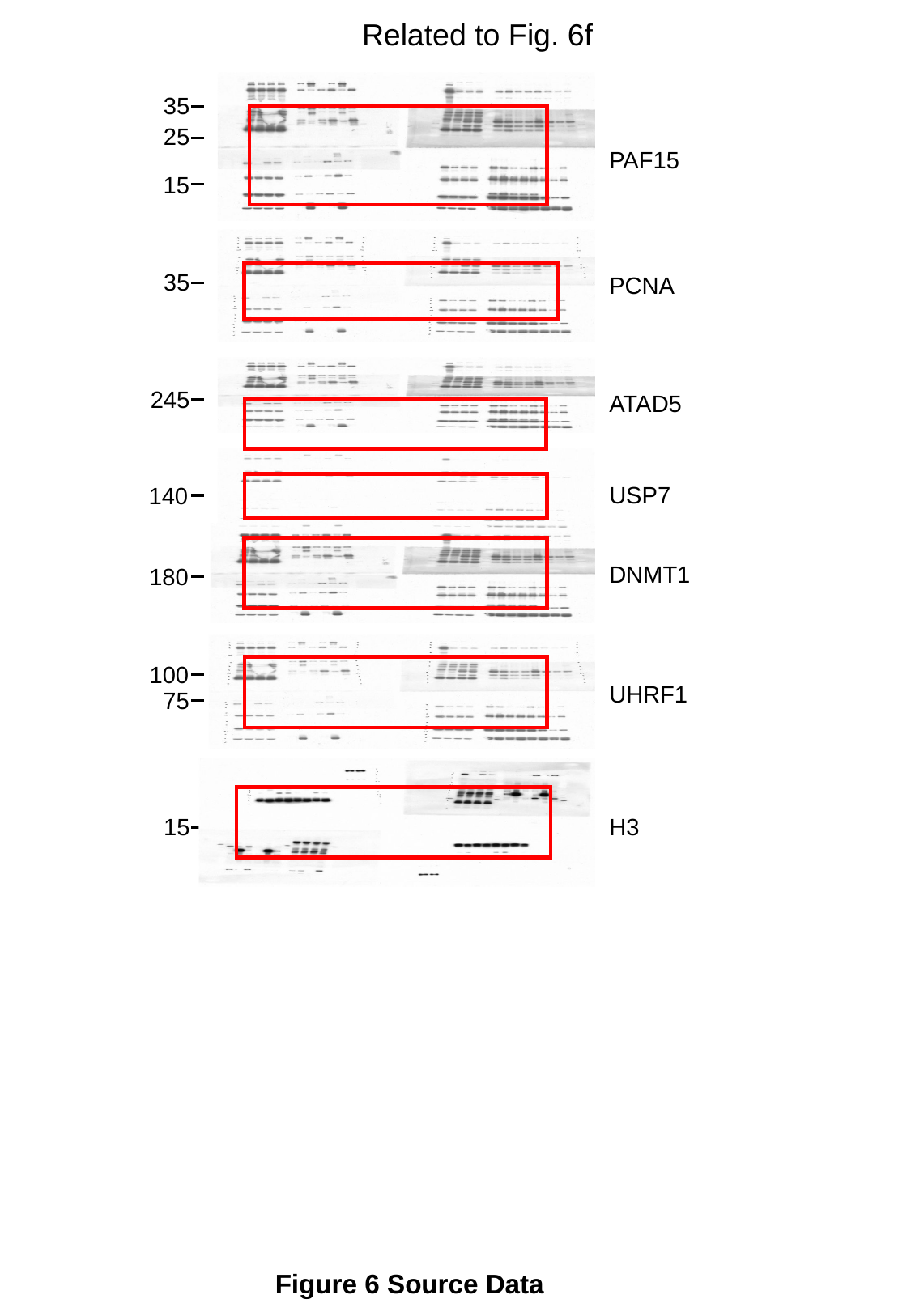

Related to Fig. 6f
35
25
PAF15
15
35
PCNA
245
ATAD5
USP7
140
DNMT1
180
100
UHRF1
75
H3
15
Figure 6 Source Data
