# Supplementary material for: The termination of UHRF1-dependent PAF15 ubiquitin signaling is regulated by USP7 and ATAD5
Source: eLife. 2023 Feb 3;12:e79013. doi: 10.7554/eLife.79013 (PMC9943068; doi:10.7554/eLife.79013)
Supplement: Figure 7—figure supplement 1—source data 1. [file elife-79013-fig7-figsupp1-data1.zip › Figure 7-figure supplement 1-source data/Figure7- figure supplement 1A&D-Source Data.pptx]

## Slide 1
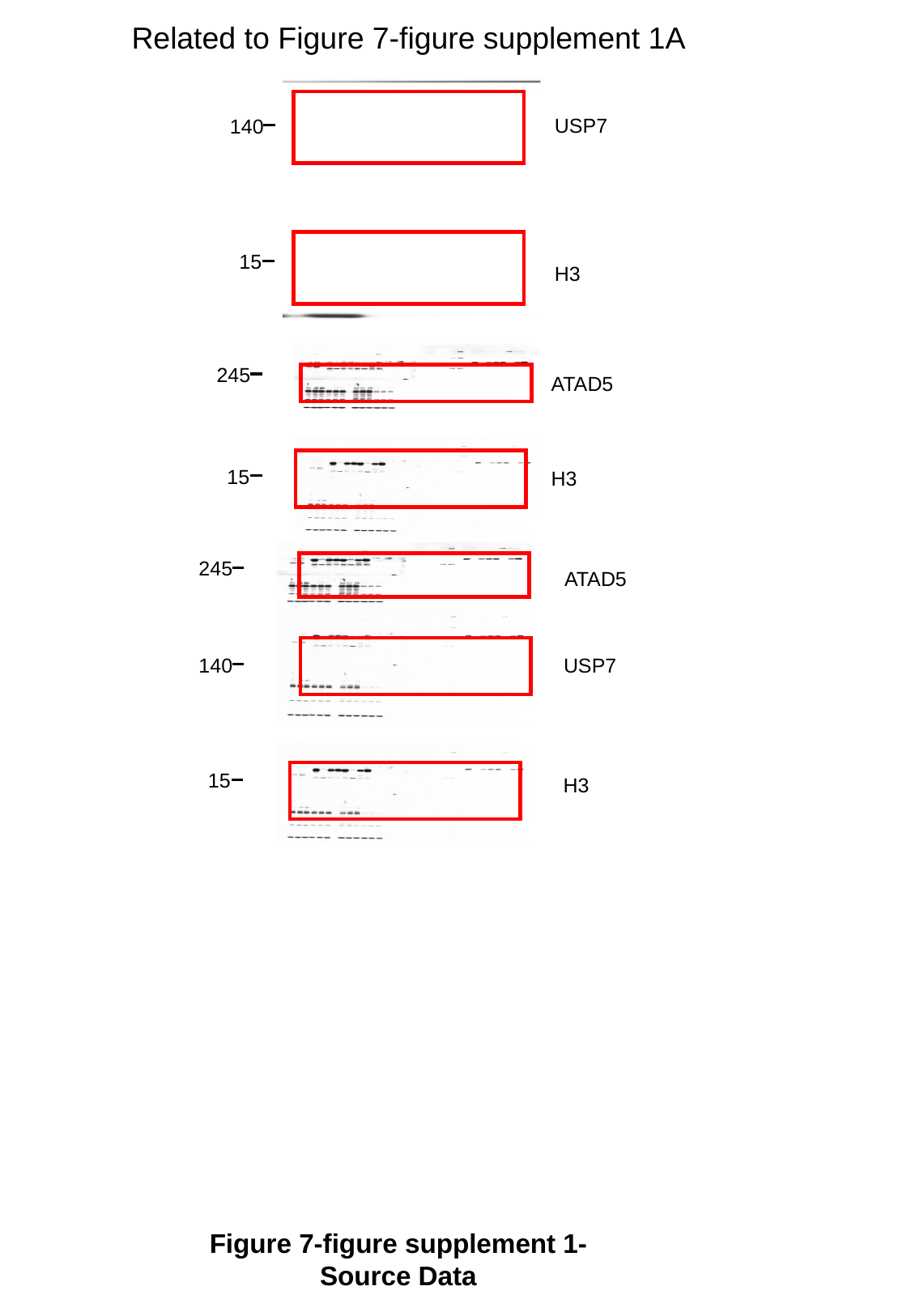

Related to Figure 7-figure supplement 1A
USP7
140
15
H3
245
ATAD5
15
H3
245
ATAD5
140
USP7
15
H3
Figure 7-figure supplement 1-Source Data

## Slide 2
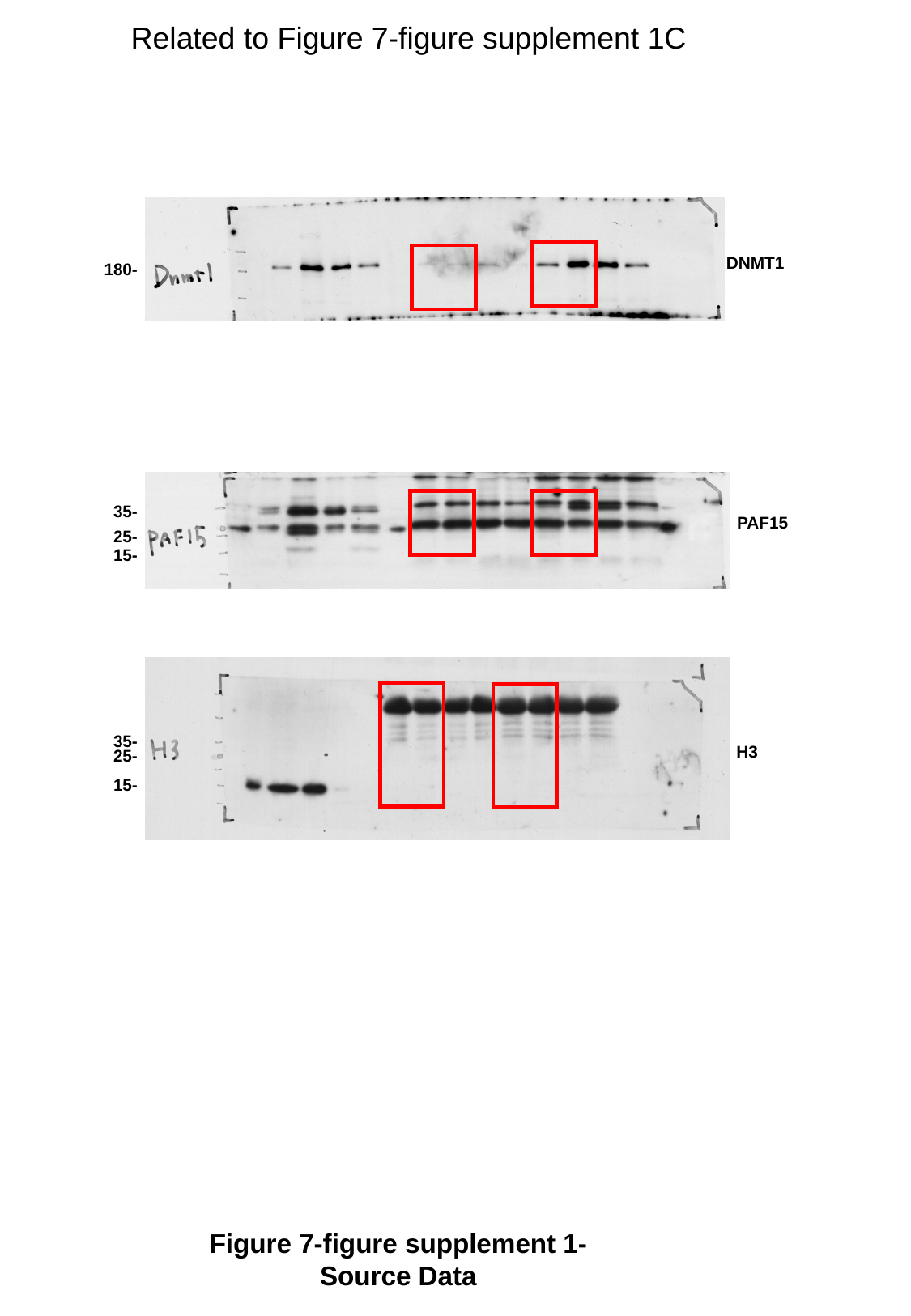

Related to Figure 7-figure supplement 1C
DNMT1
180-
35-
PAF15
25-
15-
35-
H3
25-
15-
Figure 7-figure supplement 1-Source Data
