# Supplementary material for: The termination of UHRF1-dependent PAF15 ubiquitin signaling is regulated by USP7 and ATAD5
Source: eLife. 2023 Feb 3;12:e79013. doi: 10.7554/eLife.79013 (PMC9943068; doi:10.7554/eLife.79013)
Supplement: Figure 8—source data 1. [file elife-79013-fig8-data1.zip › Figure 8-source data/Figure 8-Source Data1.pptx]

## Slide 1
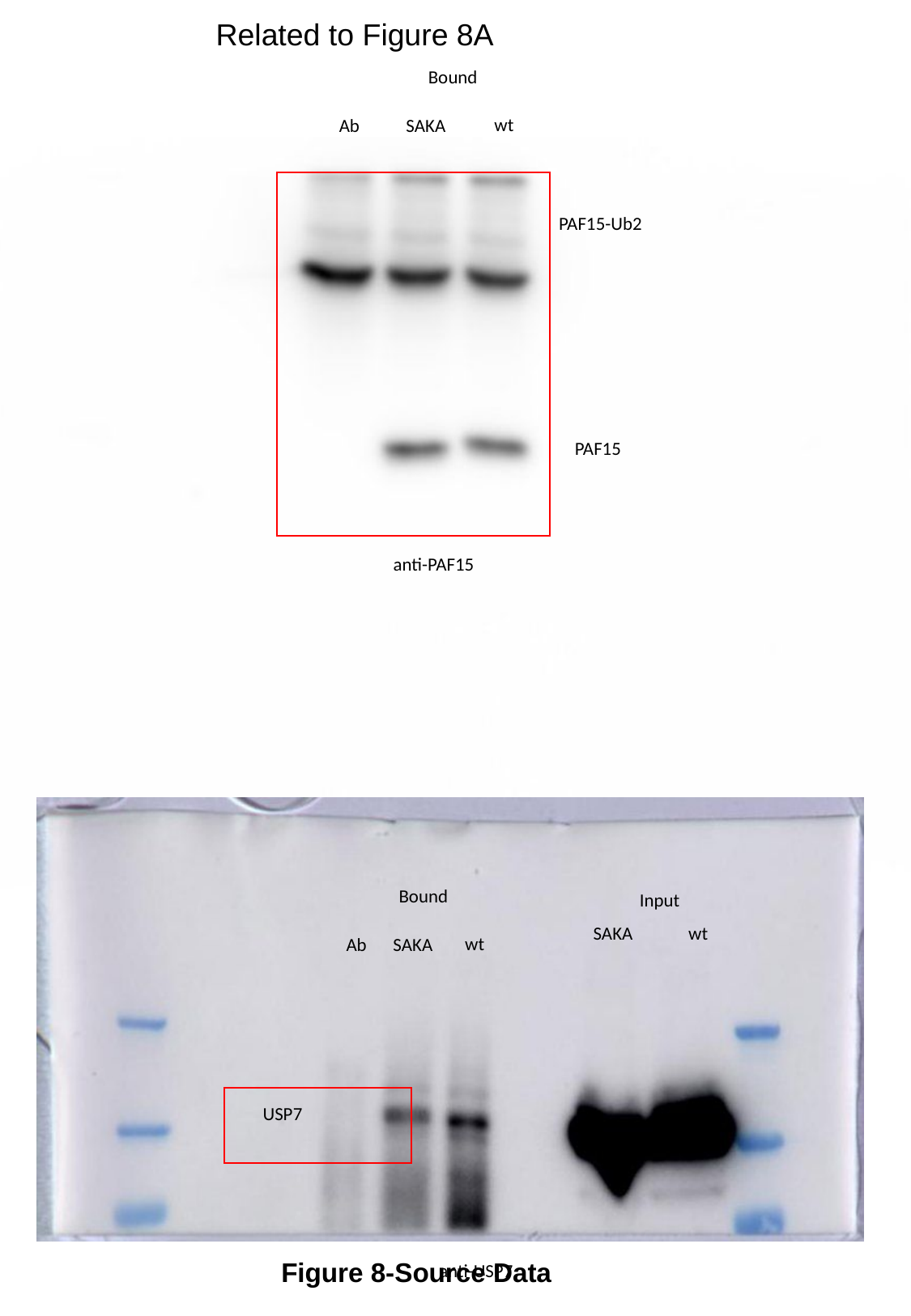

Related to Figure 8A
Bound
 wt
SAKA
Ab
anti-PAF15
PAF15-Ub2
PAF15
Bound
Input
 wt
 wt
SAKA
Ab
USP7
anti-USP7
SAKA
Figure 8-Source Data

## Slide 2
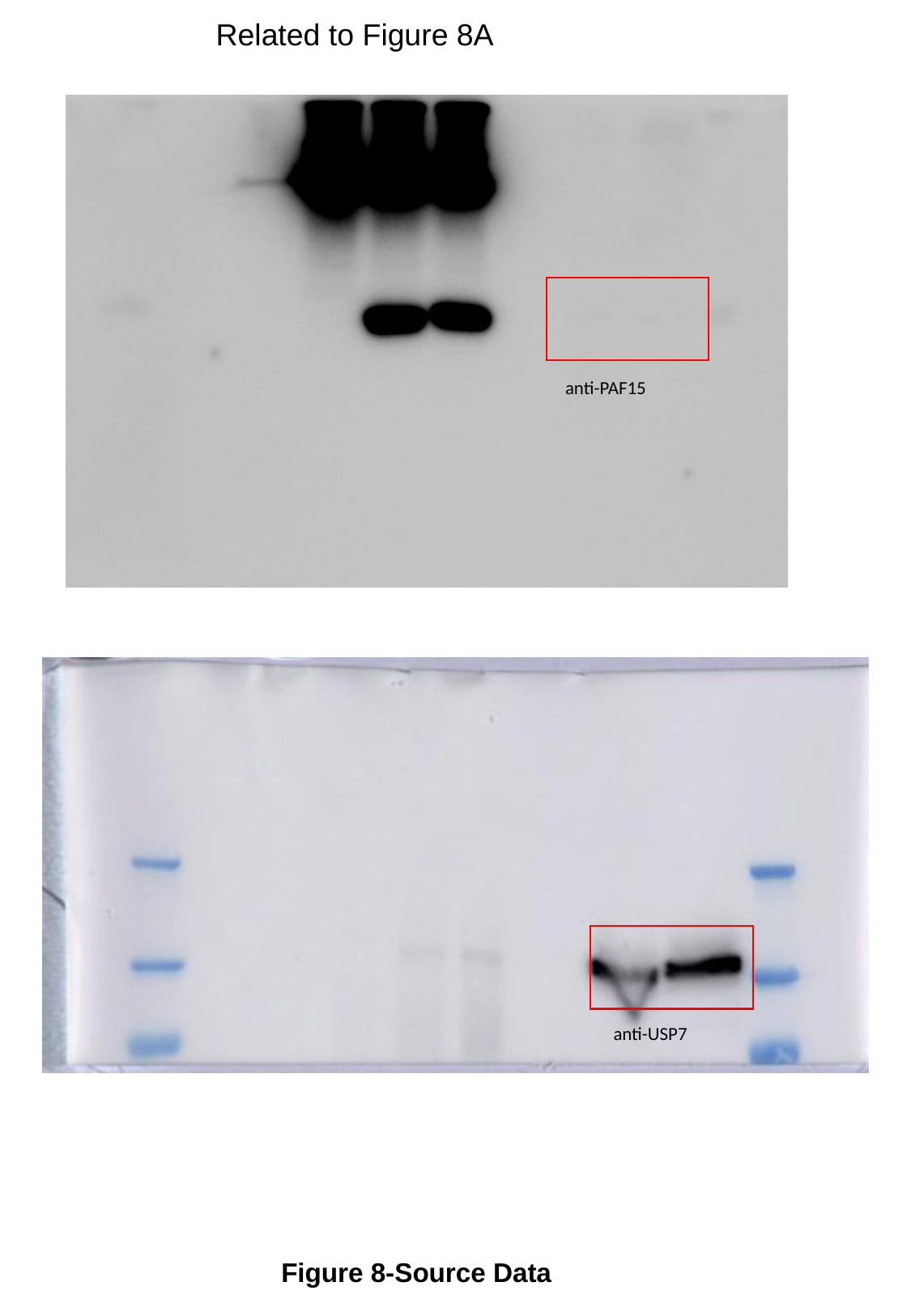

Related to Figure 8A
anti-PAF15
anti-USP7
Figure 8-Source Data

## Slide 3
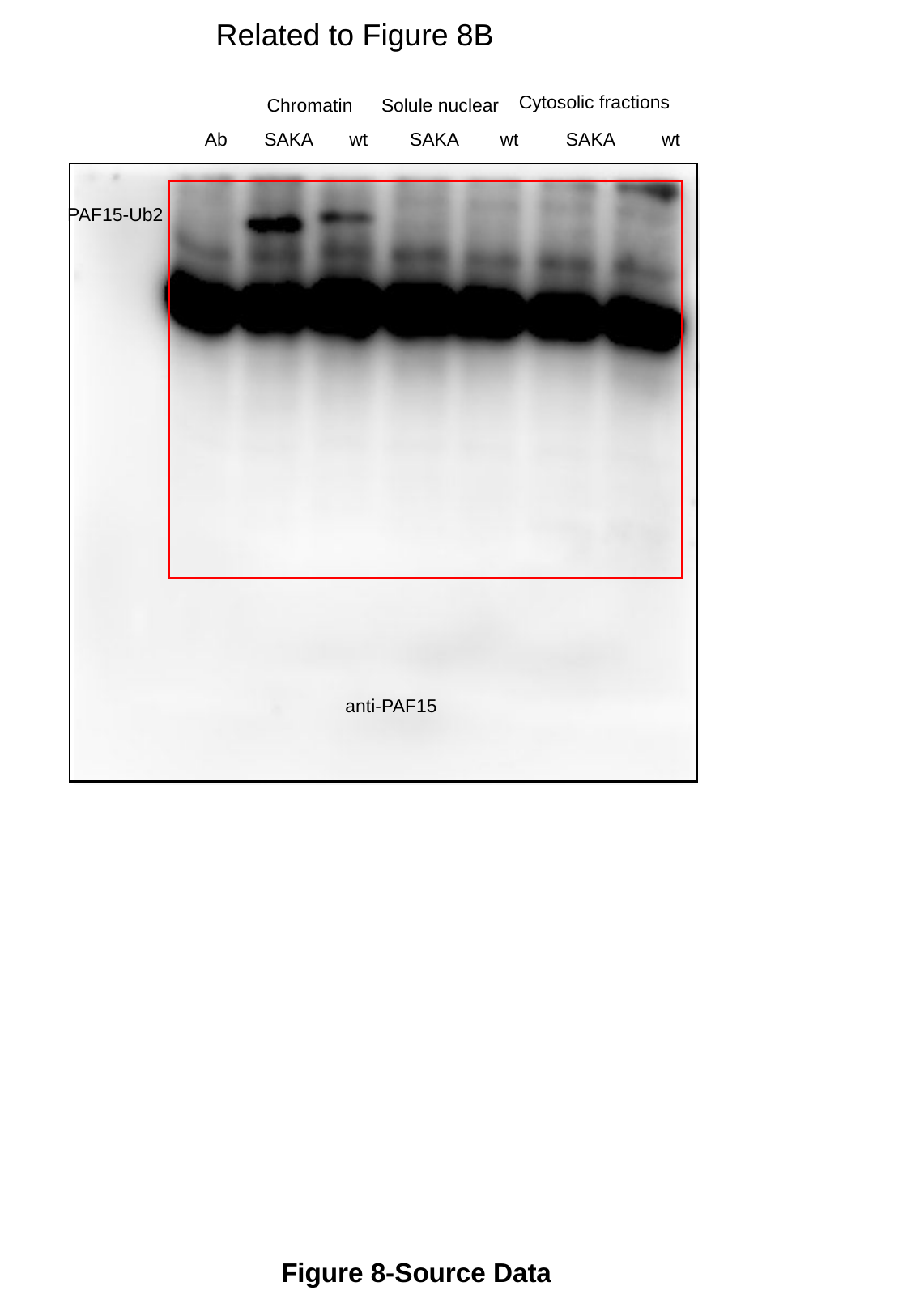

Related to Figure 8B
Cytosolic fractions
Chromatin
Solule nuclear
Ab SAKA wt SAKA wt SAKA wt
PAF15-Ub2
anti-PAF15
Figure 8-Source Data

## Slide 4
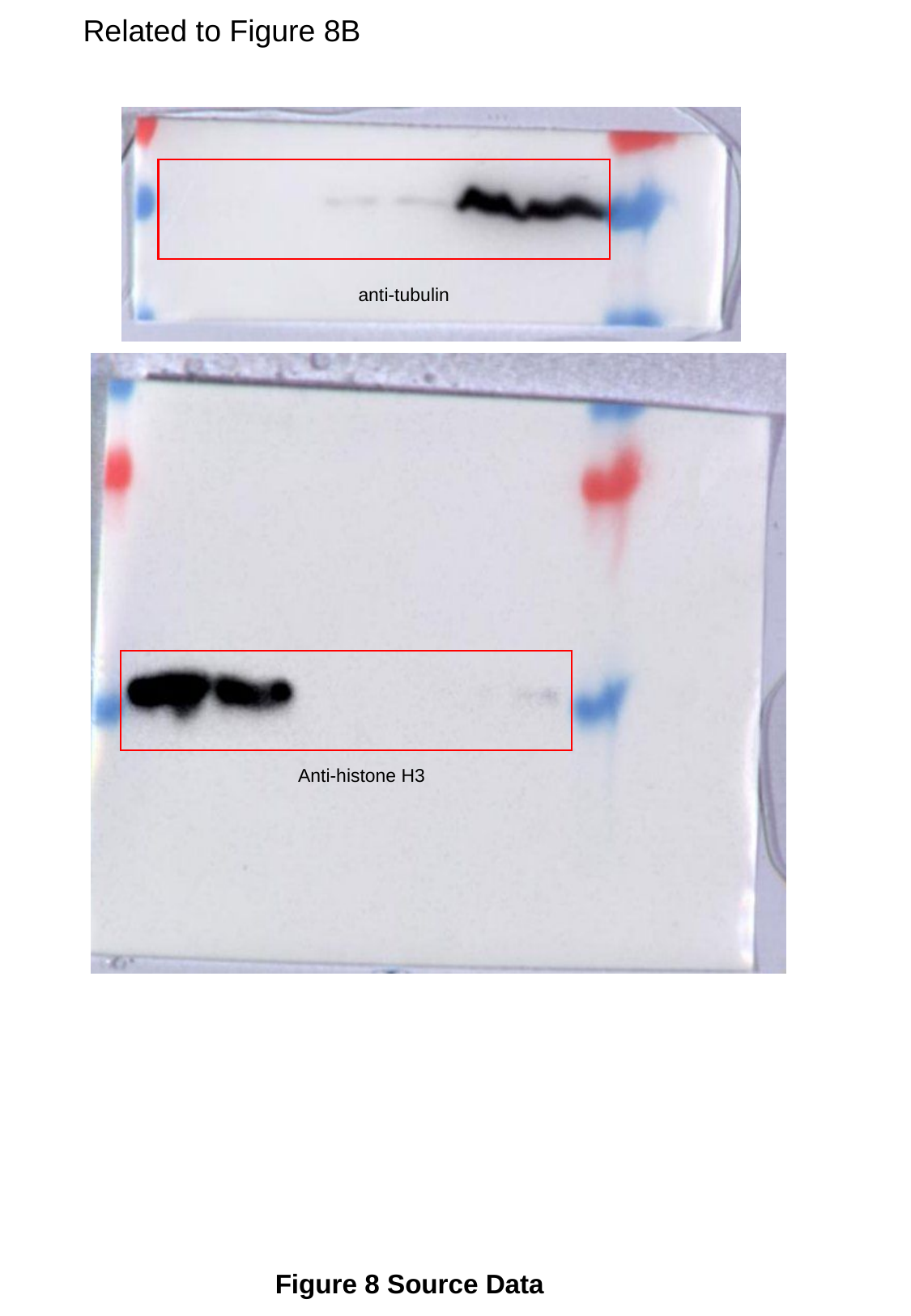

Related to Figure 8B
anti-tubulin
Anti-histone H3
Figure 8 Source Data

## Slide 5
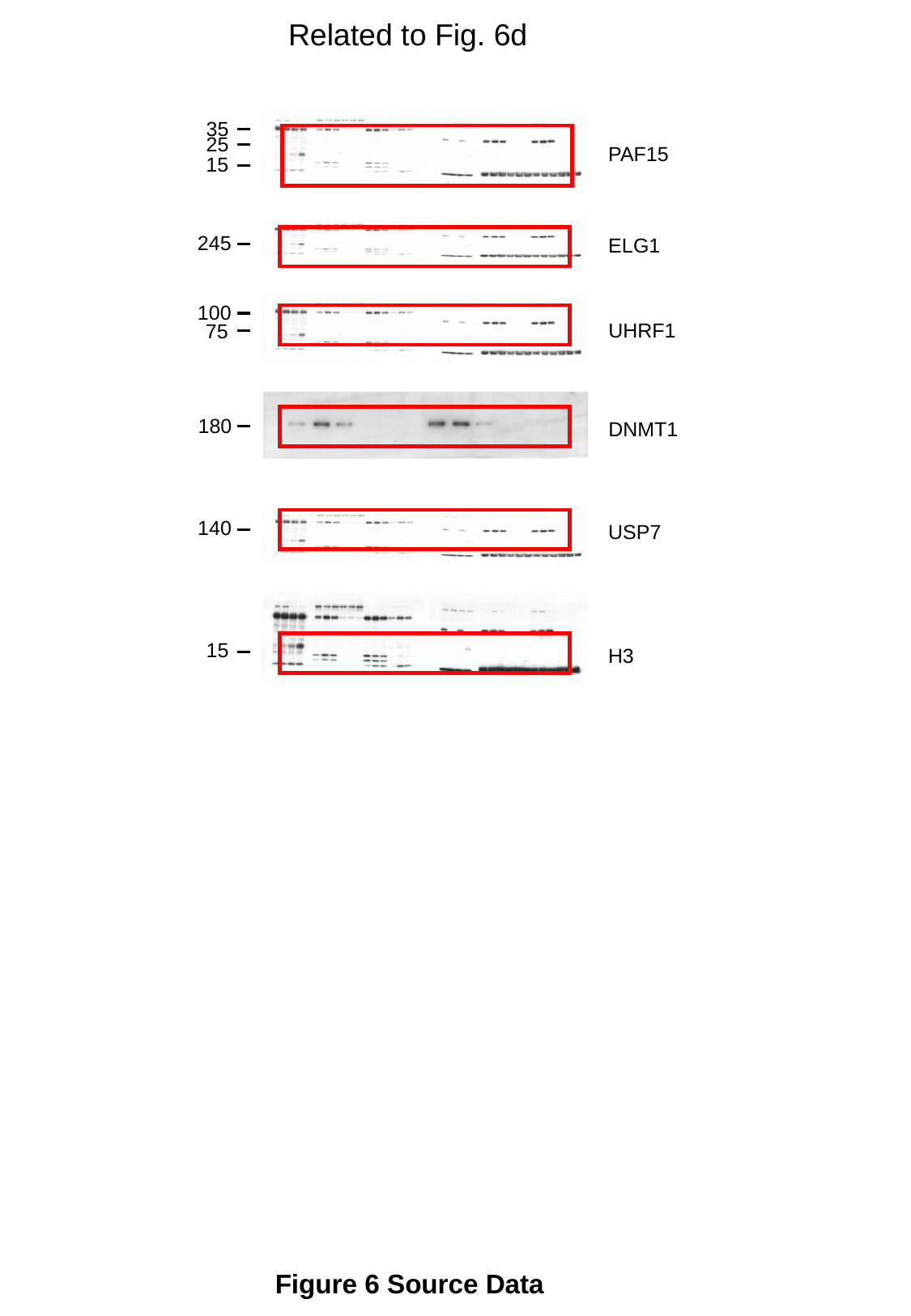

Related to Fig. 6d
35
25
PAF15
15
245
ELG1
100
UHRF1
75
180
DNMT1
140
USP7
15
H3
Figure 6 Source Data

## Slide 6
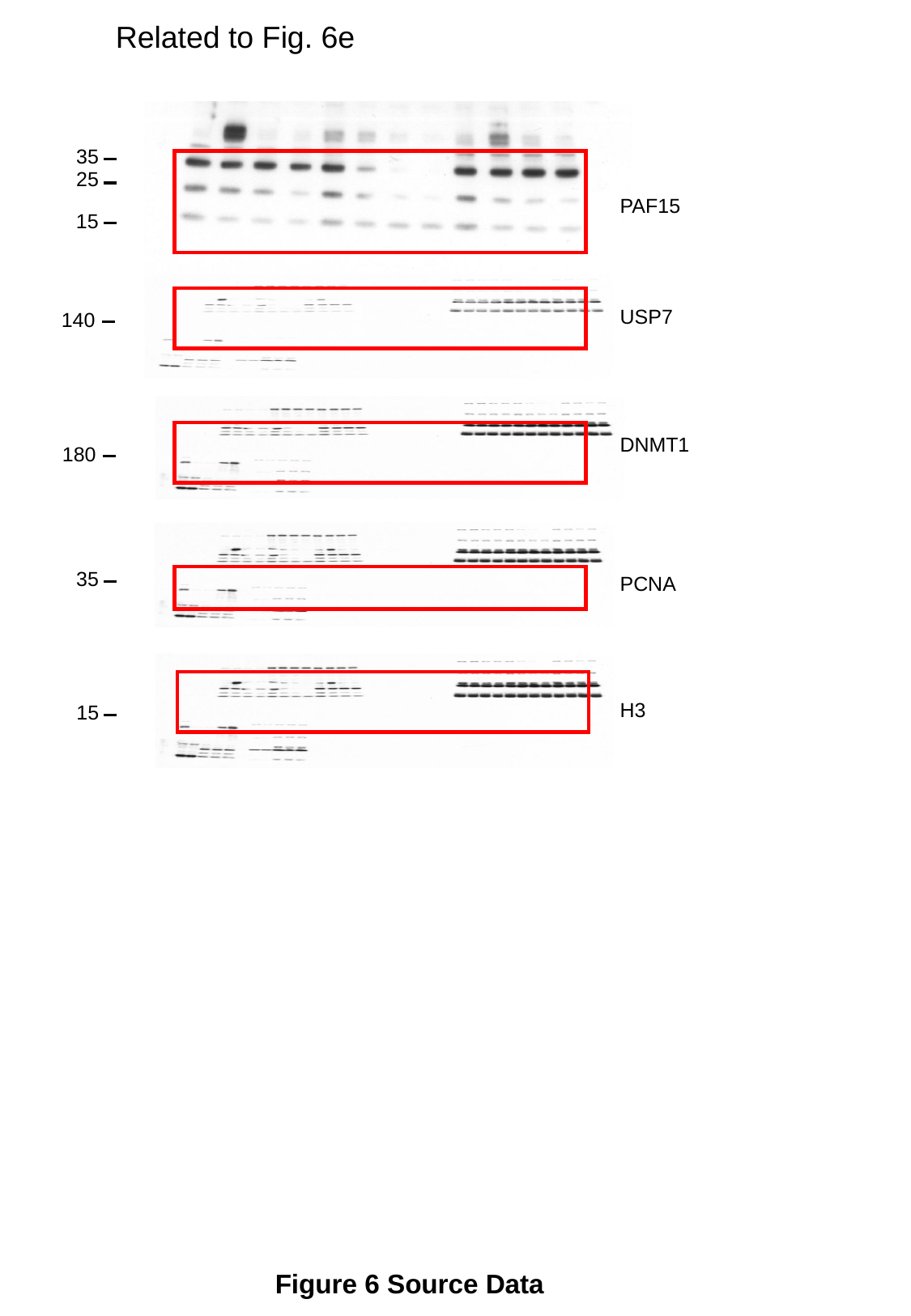

Related to Fig. 6e
35
25
PAF15
15
USP7
140
DNMT1
180
35
PCNA
H3
15
Figure 6 Source Data

## Slide 7
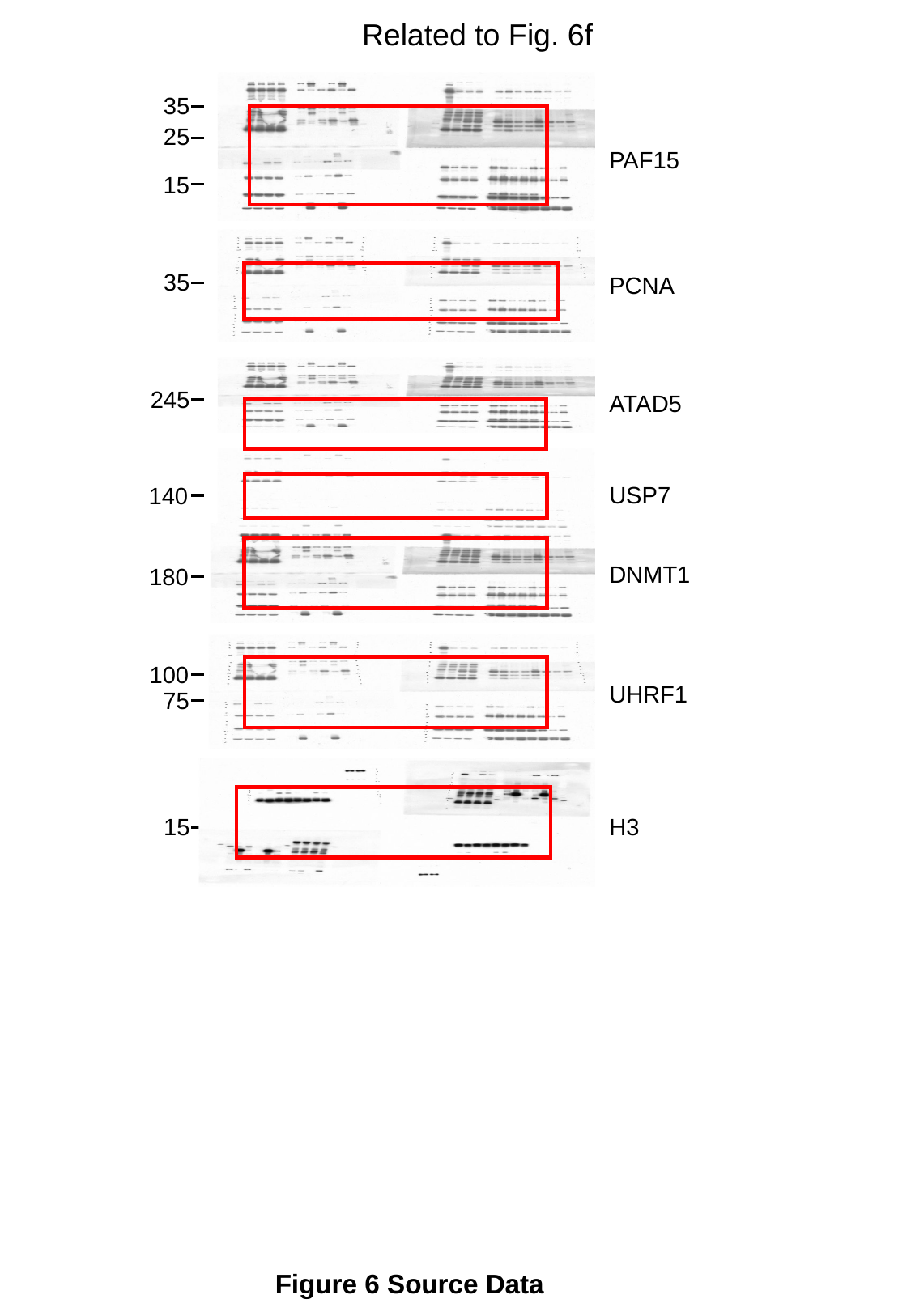

Related to Fig. 6f
35
25
PAF15
15
35
PCNA
245
ATAD5
USP7
140
DNMT1
180
100
UHRF1
75
H3
15
Figure 6 Source Data
