# Supplementary material for: The termination of UHRF1-dependent PAF15 ubiquitin signaling is regulated by USP7 and ATAD5
Source: eLife. 2023 Feb 3;12:e79013. doi: 10.7554/eLife.79013 (PMC9943068; doi:10.7554/eLife.79013)
Supplement: Figure 8—figure supplement 1—source data 1. [file elife-79013-fig8-figsupp1-data1.zip › Figure 8-figure supplement-source data/Figure8- figure supplement 1A-Source Data.pptx]

## Slide 1
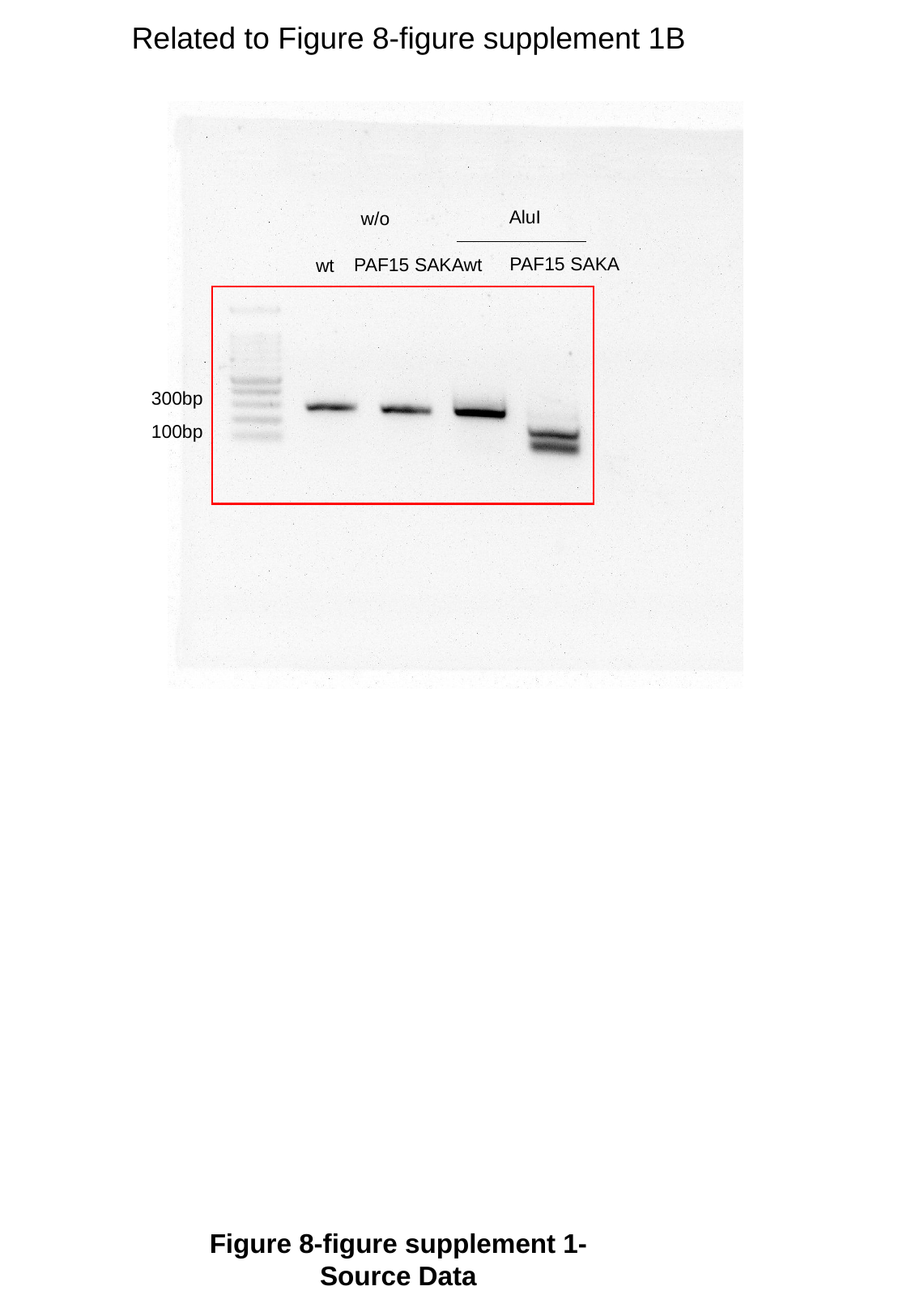

Related to Figure 8-figure supplement 1B
AluI
w/o
PAF15 SAKA
wt
PAF15 SAKA
wt
300bp
100bp
Figure 8-figure supplement 1-Source Data

## Slide 2
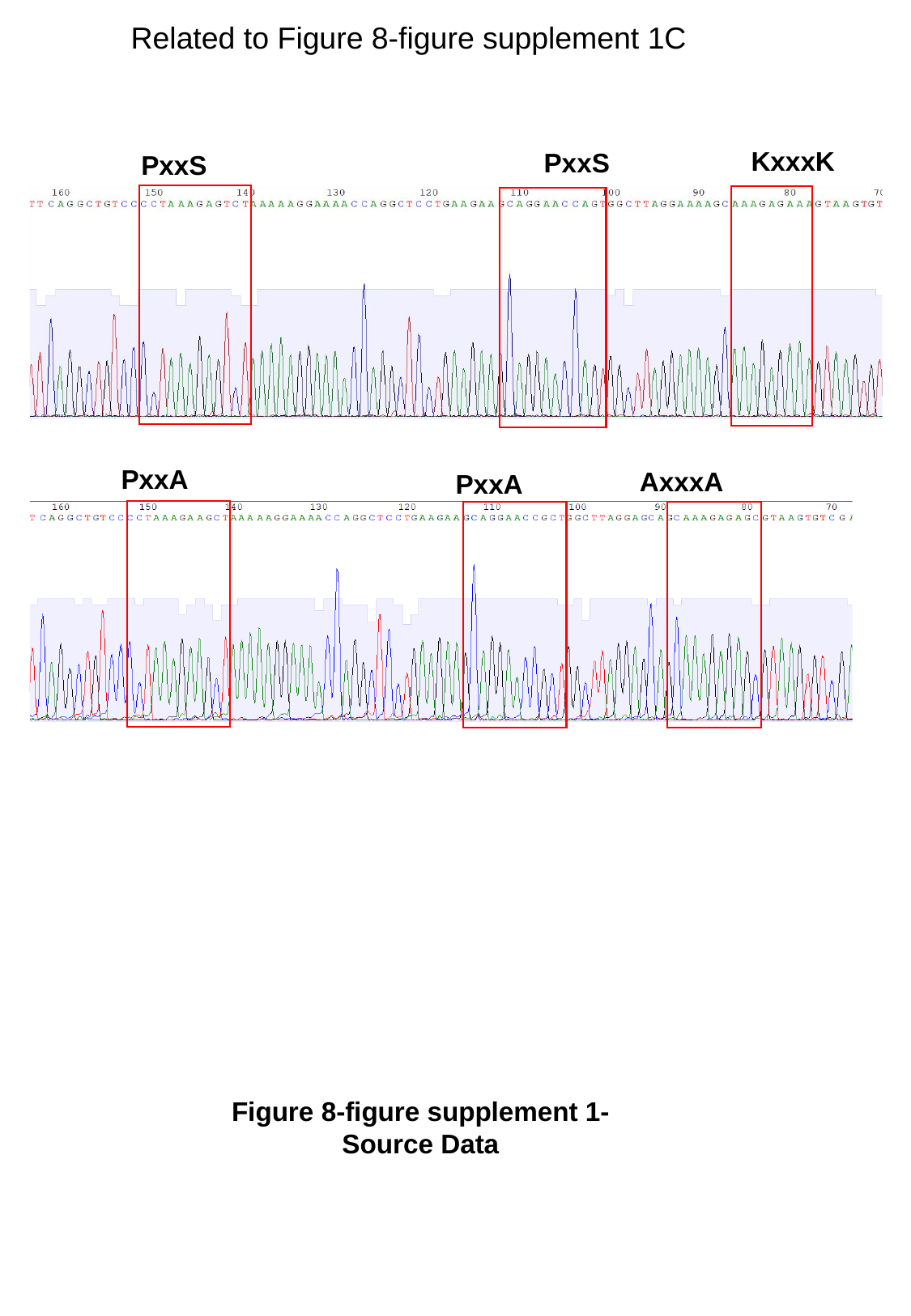

Related to Figure 8-figure supplement 1C
KxxxK
PxxS
PxxS
PxxA
AxxxA
PxxA
Figure 8-figure supplement 1-Source Data
